# Identifying information literacy skills and behaviors in the curricular competencies of health professions

**DOI:** 10.5195/jmla.2020.833

**Published:** 2020-07-01

**Authors:** Micah J. Waltz, Heather K. Moberly, Esther E. Carrigan

**Affiliations:** 1 mwaltz@tamu.edu, Lecturer, Veterinary Integrative Biosciences Department, College of Veterinary Medicine & Biomedical Sciences, and Joint Appointment to Medical Sciences Library, University Libraries, Texas A&M University, College Station, TX; 2 hmoberly@tamu.edu, Dorothy G. Whitley Professor and Coordinator of Veterinary Services, Medical Sciences Library, University Libraries, and Joint Appointment to Veterinary Integrative Biosciences Department, College of Veterinary Medicine & Biomedical Sciences, Texas A&M University, College Station, TX; 3 ecarrigan@library.tamu.edu, Professor and Deputy Director, Medical Sciences Library, University Libraries, Texas A&M University, College Station, TX

## Abstract

**Objective:**

This research identified the presence of information skill and behaviors components of information literacy in curricular competencies to inform a medical sciences library's instructional schema for five different professional programs at Texas A&M University: College of Medicine, College of Nursing, College of Veterinary Medicine and Biomedical Sciences, Irma Lerma Rangel College of Pharmacy, and School of Public Health.

**Methods:**

Curricular competency documents were collected from each program and reviewed. Coding categories were identified from the curricular competencies of professional health curricula using data-driven qualitative coding. To guide the identification and coding of competency categories, we developed a seven-category rubric from the coding categories. Three researchers used this rubric to independently code the categories of all of the included professional health curricular competencies. An additional researcher used a revised version of the rubric to identify action verbs in each competency.

**Results:**

Competencies for four of the five professional health curricula explicitly stated information skills and behaviors. Each of the five curricula included several competencies that depended on information-specific skills and behaviors. The most common verb used to describe implicit or explicit competencies was “evaluate.”

**Conclusions:**

The representation of information skills and behaviors aligns with the drive behind the Association of College & Research Libraries (ACRL) Framework for Information Literacy for Higher Education. Both underpin the importance of evidence-based medicine methodology.

## INTRODUCTION

Librarians and information specialists provide a variety of resources, services, and instruction to stakeholders. Stakeholder instruction requests range from one-shot instruction to coordination throughout a curriculum. Librarian instruction and assignment support may benefit from increased curricular coordination in programs emphasizing evidence-based practice (EBP), such as professional health programs. Engagement of librarians permits scaffolding information skills and behaviors throughout an entire professional curriculum [1].

Information skills and behaviors are implied as part of other program curricular competencies, such as lifelong learning or EBP [2]. The foundational nature of information skills and behaviors is reflected in the *Medical Library Association (MLA) Competencies for Lifelong Learning and Professional Success* [3]. In 2016, the Association of College & Research Libraries (ACRL) of the American Library Association (ALA) published the *Framework for Information Literacy for Higher Education* (ACRL Framework) to update the previous *Information Literacy Competency Standards for Higher Education* [4]. The ACRL Framework has been designed to provide support and guidance to librarians about teaching information literacy through a loose structure of six interconnected core concepts. Additionally, some discipline-specific standards for information literacy also exist through ACRL, such as nursing education [5]. These frameworks and discipline-specific standards can guide instruction about information skills and behaviors for professional program curricula. However, frameworks and standards, like the ACRL Framework and ACRL Information Literacy Competencies for Nursing, are often difficult to match to program curricular competencies because program competencies often only imply information skills or do not emphasize them at all.

However, professional health sciences curricula are emphasizing EBP and moving toward competency-based education [6, 7], both nationally and internationally. Human medicine [8] and veterinary medicine [9] both embrace EBP, which is generally taught as a five-step process: “Ask,” “Acquire,” “Appraise,” “Apply,” and “Assess” [10]. Librarians and information specialists can support several steps of this process, depending in part on their training and experience; however, the Acquire step is squarely in the domain of librarianship. In the Acquire step, students are first taught to search for evidence using indexes, databases, and other resources as appropriate to the topic. Students then identify the evidence that is appropriate for the topic and obtain the full text. For the purposes of this study, library instruction for EBP, evidence-based medicine (EBM), and evidence-based veterinary medicine (EBVM) is sufficiently similar that the topics may be considered synonymous.

The Medical Sciences Library (MSL) at Texas A&M University supports health sciences programs at the university's flagship campus in College Station and four other locations, serving as their primary library. The following professional degree programs are supported: doctor of medicine (MD) [11], two masters' of nursing (MSN/MSN family nurse practitioner) [12], doctor of veterinary medicine/veterinarian (DVM) [13], doctor of pharmacy (PharmD) [14], and the master's and doctor of public health (MPH/DPH) [15]. The purpose of this study was to explore whether program-level curricular documents for five professional health degree programs at Texas A&M could yield data to inform the development of a master MSL instruction schema. This schema will be used to provide equitable support to all the MSL-supported health professional programs. To address this objective, curricular documents were examined from the five MSL-supported professional programs: medical, nursing, pharmacy, public health, and veterinary medicine.

## METHODS

### Data collection

In October 2017, program-level curricular documents were collected for the five MSL-supported professional schools: College of Medicine (Medical), College of Nursing (Nursing), College of Veterinary Medicine & Biomedical Sciences (CVM), Irma Lerma Rangel College of Pharmacy (Pharm), and School of Public Health (PHEB). The competencies for Medical, Nursing, and Pharm were openly accessible and found through an Internet search. Liaison librarians for PHEB and CVM provided their professional programs' curricular competencies.

### Data analysis software

The qualitative data analysis software, MAXQDA, was used for coding [16]. MAXQDA is a mixed-methods software that includes built-in statistical analyses; textual data coding capabilities; and a robust data collector that captures web pages, computer files, and videos. We received portable document format (PDF) copies of the curricular documents from all five professional programs (supplemental Appendixes A–E). Before coding the Texas A&M professional programs' curricular documents, we underwent training on how to use the coding functions in MAXQDA using openly available curricular competencies from other universities' health sciences programs.

### Qualitative coding

Data-driven qualitative coding was used to analyze the curricular documents from the five professional programs [17]. This inductive approach allowed competencies to be categorized [18]. Six categories were initially identified from the five programs' competencies, and an “other” category was included to ensure that all competencies could be coded.

### Coding frame and rubric development

Differences between competencies were often subtle, such as the difference between “knowing risk factors” and “using risk factors to create a differential diagnosis.” The process of sentence diagramming was modified as an approach to differentiate between competencies with slightly different emphases. Using this concept of sentence diagramming, the action verb of the sentence was used to identify the overall purpose of the competency.

Creating a coding frame was the first step of inductive qualitative coding. This coding frame was created by identifying seven coding categories from the five professional programs' curricular documents. In the second step, the coding frame was used to develop a coding rubric to code the five professional programs' curricular documents (supplemental Appendix F). Generating a coding frame from the five professional programs' curricular documents that applied equally to all the documents was a data-driven approach. This data-driven approach emphasized internal consistency and generated a custom coding frame and subsequent custom coding rubric that was appropriate for, and consistent with, the documents to be coded. External bias from a preexisting coding frame with less applicable categories was avoided by creating the coding frame and the coding rubric from the curricular documents themselves.

We used the coding frame to develop a coding rubric that served as a working document when coding the professional programs' curricular documents. The rubric began with a question that prompted identification of the overarching category (Table 1). The rubric then moved to inclusion and exclusion criteria. The inclusion criteria had a partially diagrammed example sentence with common types of direct objects for the specific category. The differences between gaining and applying knowledge were distinguished using exclusion criteria. Each of us provided feedback on an initial draft of the coding rubric and identified anything unclear, including instructions. The rubric was updated based on this feedback.

**Table 1:** Example of coding rubric

Preliminary code categories	Coding question	Inclusion criteria	Exclusion criteria	Exclusion criteria example
Clinical Skills	Is *treatment* OR *diagnosis* OR *providing care* the overarching theme of the competency?	[action verb]+description about or use of medical tests/procedures, development of patient plans, physical examinations/palpations, use of collected information/data to inform diagnosis, and phrases such as “provide health OR palliative care.”	If the medical tests or procedures are NOT the focus and are being used FOR an application that would be categorized in a different core area	Demonstrate understanding of epidemiology of common diseases within a population and the approaches which are useful in reducing their incidence and prevalence *TAMU Medical School Competencies

The rubric was developed to help researchers efficiently and consistently categorize each competency. The inclusion criterion was a partially diagrammed sentence that focused the coder on the action verb of the competency with examples of common direct objects. Exclusion criteria were included to help the coders distinguish between differences in gaining skills and applying them. Examples were included for the exclusion criteria to further help the coders distinguish among competency categories.

### Qualitative coding

Each researcher used the updated coding rubric to qualitatively code each competency for its overarching category using a data-driven approach. Only a single code could be assigned per curricular competency. We met after independently coding the 5 curricular documents. Initial agreement in coding categories was 61.9% before discussion and 88.4% after discussion; therefore, inter-rater reliability statistics were not calculated. If consensus was not reached through discussion, a fourth researcher who did not participate in coding arbitrated.

### Analytic coding of verbs

While comparing our independent coding, we noted that the coded overarching theme did not account for nuances in the written documents in some curricular competencies. To account for these nuances, a second round of coding focused on which skills were implied by the verbs within the competency. Another researcher who did not participate in the qualitative coding performed this analytic coding of verbs. This approach avoided any bias that may have developed among the original three researchers during qualitative coding. The additional researcher was tasked with using verbs to split apart each individual curricular competency into phrases. Each of these phrases was then coded using the rubric generated for qualitative coding. The nuances implied by various clauses and phrases within a competency were accounted for by analytically coding the parts of each competency. The clauses and phrases were used to categorize the implied, rather than explicit, information skills and behaviors activities in curricular competencies.

## RESULTS

**Figure 1:**
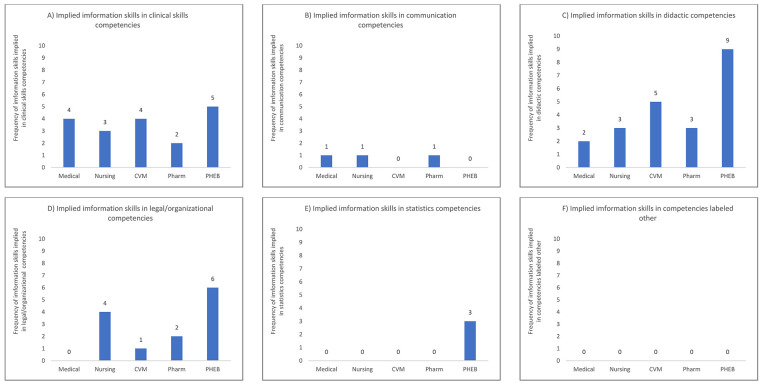
Co-occurrences of implied competencies occurring in the 6 coding categories other than information skills and behaviors for the professional program curricular documents

From the five colleges' professional programs' curricular documents, we identified seven generalizable categories: clinical skills, communication, didactic knowledge, information skills and behaviors, legal, statistics/experimental design, and other (Table 2). Few explicit statements about information skills or behaviors were apparent among the five programs' curricular documents. However, multiple competencies in all five of the programs' curricular documents required students to demonstrate skills that required information skills and behaviors. Of the five professional programs, four had one to two curricular competencies that explicitly concerned information skills and behaviors. However, all five had multiple competencies that implicitly concerned information skills and behaviors (Figure 1).

**Table 2:** Competencies with implicit or explicit information skills and behaviors

College	Competency	Coded phrase	Active verbs from coded phrase	Reason for code	Analytic coding of verbs	Implicit/explicit
College of Medicine (Medicine)	Develop contextual and individualized diagnostic and treatment plans based on collected clinical information	Develop…plans… based on collected clinical information	Based	This involves an analysis of the collected information to formulate treatment plans; the analysis of the information is an example of information skills and behaviors.	Evaluating	Implicit
Medicine	Interpret the results of commonly used laboratory and radiologic studies	Interpret the results of…	Interpret	This is an evaluation of collected data; the interpretation of data is an example of information skills and behaviors.	Evaluating	Implicit
Medicine	Maintain accurate medical records	Maintain accurate medical records	Record	Maintaining accurate medical records involves evaluating the information on hand to make sure it is correct, then seeking out new information if it is not.	Evaluating	Implicit
Medicine	Properly utilize clinical, laboratory, radiologic, and pathological examinations to diagnose and treat common maladies	Properly utilize clinical, laboratory, radiologic, and pathological examinations…	Utilize	The phrase is talking about properly using various examinations for the ultimate purpose of making a diagnosis. This would mean the given information needs to be evaluated and contextualized within current research.	Searching, evaluating, other	Implicit
Medicine	Select, appraise, and utilize evidence from scientific studies related to clinical questions and patients' health problems	Select, appraise, and utilize evidence from scientific studies	Select, appraise, utilize	The selecting, appraising, and utilizing evidence from scientific studies is about information skills and behaviors. This includes three elements of information skills and behaviors: searching for information, evaluating any information, and then applying it.	Searching, evaluating, other	Explicit
Medicine	Utilize information resources and available data to support lifelong learning	Utilize information…	Utilize	This competency requires students to update their knowledge using information (lifelong learning).	Other	Explicit
College of Nursing (Nursing)	Analyze the links among practice, organizational, population, fiscal, and policy issues to advocate for improved patient outcomes.	Analyze the links among practice, organizational, population, fiscal, and policy issues…	Analyze	This is an evaluation of information from multiple sources to improve patient care.	Evaluating	Implicit
Nursing	Demonstrate advanced levels of clinical judgment, systems thinking, and accountability in designing, delivering, and evaluating evidence-based care to improve patient outcomes.	…Evaluating evidence-based care…	Evaluating	This is an evaluation of given information, both clinical and current research, for improving patient outcomes.	Evaluating	Implicit
Nursing	Design, implement, and evaluate therapeutic and preventative interventions based on nursing science, and other sciences and humanities.	…Evaluate therapeutic and preventative interventions…	Evaluate	This is an evaluation of scientific literature for application purposes.	Evaluating	Implicit
Nursing	Integrate organizational, client centered, and culturally centered approaches to plan, deliver and evaluate health care for individuals, families and populations.	…Evaluate health care for individuals, families and populations.	Evaluate	This is an evaluation of information to develop a treatment plan.	Evaluating	Implicit
College of Veterinary Medicine (CVM)	Construct an appropriate diagnostic plan, based upon their preliminary differential diagnosis list, and analyze the findings in order to reach a presumptive diagnosis of common medical conditions in common domestic species	…Analyze the findings…	Analyze	Analyzing clinical information includes contextualizing clinical data with current research.	Evaluating	Implicit
CVM	Evaluate risk factors for individual or groups of animals for medical conditions in common domestic species	Evaluate risk factors…	Evaluate	This is an evaluation of clinical information and relating it to current published reference values.	Evaluating	Implicit
CVM	Evaluate the scope of their personal and professional limits and appropriately judge when to seek professional advice, assistance, and support and manage a referral case	Evaluate the scope of their personal and professional limits…	Evaluate	This form of self-evaluation implies information-seeking behavior because professional limitations have published guidelines and expected behaviors that an individual must be able to compare to.	Evaluating	Implicit
CVM	Identify, review, and critically evaluate biomedical literature and apply it to the practice of contemporary, evidence-based veterinary medicine	…Critically evaluate biomedical literature…	Evaluate	This is an evaluation of scientific literature for application purposes.	Evaluating	Explicit
CVM	Recognize the need for sedation, anesthesia, and analgesia and evaluate the pertinent patient information in order to formulate an appropriate anesthesia protocol in common domestic species	…Evaluate the pertinent patient information…to…	Evaluate	This an evaluation of given information to develop appropriate protocols.	Evaluating	Implicit
CVM	Select appropriate surgical interventions, based upon the identification of pathologic conditions and the evaluation of patient conditions, to treat surgical problems in common domestic species	…Evaluation of patient conditions…	Evaluation	Identification of pathological conditions includes knowing about various types of pathological conditions and staying updated with the current literature as new ones are published about or new categorizations are developed.	Evaluating	Implicit
Irma Lerma Rangel College of Pharmacy (Pharm)	Access, analyze, and interpret relevant resources to provide comprehensive drug information to patients and health care providers on the safe and effective use of medications	Access, analyze, and interpret relevant resources…	Access, analyze, interpret	This is an analysis of the given information, which is a hallmark of information-seeking behavior and the combination falls under searching (Access) and evaluation (Analyze/Interpret) of relevant sources	Searching, evaluating	Explicit
Pharm	Maintain professional competence by identifying and evaluating emerging issues, trends, products, and services that may impact patient and population outcomes, management of resources and medication use systems, disease prevention services and public health policy	Identifying and evaluating emerging issues, trends, products, and services…	Identify, evaluate	Both identifying and evaluating the emerging information is coded under information-seeking behavior.	Identifying, evaluating	Implicit
Pharm	Provide evidence-based care to populations through disease management programs and protocols that are derived from analysis of epidemiologic and pharmacoeconomic data, medication use criteria, medication use review, and risk reduction strategies	…Analysis of epidemiologic and pharmacoeconomic data, medication use criteria, medication use review, and risk reduction strategies	Analysis	This is an evaluation of information from multiple sources to provide evidence-based care.	Evaluating	Implicit
School of Public Health (PHEB)	Apply basic informatics techniques with vital statistics and public health records in the description of public health characteristics and in public health research and evaluation.	Apply basic informatics techniques with…statistics…and…records…in public health research and evaluation.	Evaluation	An inherent element of this competency is evaluating sources of information	Evaluating	Implicit
PHEB	Apply ethical principles to public health program planning, implementation and evaluation	Apply ethical principles to…evaluation	Apply, evaluation	This focuses on the ethical elements involved with creating and evaluating public health programming. The information skills and behaviors element is how ethics are a part of evaluating public health programming.	Evaluating	Implicit
PHEB	Apply evidence-based approaches in the development and evaluation of social and behavioral science interventions	Apply evidence-based approaches in the development and evaluation.	Apply, evaluation	This phrase is about using evidence-based approaches for interventions. The application falls in the evaluation element.	Evaluating	Implicit
PHEB	Apply the core functions of assessment, policy development, and assurance in the analysis of public health problems and their solutions	…The analysis of public health problems and their solutions	Analysis	This competency is about using various didactic elements to analyze public health problems and solutions. The analysis element is about evaluating problems and their solutions.	Evaluating	Implicit
PHEB	Describe steps and procedures for the planning, implementation, and evaluation of public health programs, policies, and interventions.	…Evaluation of public health programs	Evaluate	This element of the competency has to do with an evaluation of the given information; the intention is explaining how to evaluate specified information.	Evaluating	Implicit
PHEB	Describe the principles of program planning, development, management, and evaluation in public health initiatives	Describe… evaluation in public health initiatives	Evaluation	The evaluation of public health initiatives must be described; the information skills piece is about being able to evaluate public health initiatives.	Evaluating	Implicit
PHEB	Identify key sources of data for epidemiologic purposes	Identifying…data	Identify	This is about identifying different data for a specific purpose.	Identifying	Explicit
PHEB	Interpret results of statistical analyses found in public health studies	Interpret results…	Interpret	Interpreting results is the application of information for a purpose, which falls under evaluation in information-seeking behavior.	Evaluating	Implicit
PHEB	Draw appropriate inferences from epidemiologic data.	Draw appropriate inferences…	Inference	Drawing inferences from information is a type of information-seeking behavior.	Evaluating	Implicit
PHEB	Evaluate the strengths and limitations of epidemiologic reports.	Evaluate the strengths and limitations of epidemiologic reports	Evaluate	This is about evaluating the information in specific sources of data.	Evaluating	Implicit
PHEB	Identify critical stakeholders for the planning, implementation, and evaluation of public health programs, policies, and interventions	…Evaluation of…	Evaluate	This is about identifying the stakeholders necessary for developing and evaluating various types of interventions. Evaluating the various sources of data falls under information-seeking behaviors.	Evaluating	Implicit

Curricular documents with competencies had either explicitly or implicitly stated information skills and behaviors. These competencies were coded, and the specific elements of information skills and behaviors identified, including the verbs used for analytic coding.

The number of total competencies varied by program.

Medical=College of Medicine, Nursing=College of Nursing, CVM=College of Veterinary Medicine & Biomedical Sciences, Pharm=Irma Lerma Rangel College of Pharmacy, and PHEB=School of Public Health.

### Language explicitly describing information skills and behaviors

Competencies explicitly stating information skills or behaviors were described in a variety of ways. A program's expectations of their students' abilities with information skills and behaviors could be derived from the about which ways these skills and behaviors were written about. A striking example of a difference between health professions is demonstrated by separate Medical and Pharm competencies about information skills and behaviors.

The Medical competency—“Utilize information resources and available data to support lifelong learning” [11]—explicitly states that medical students must demonstrate the ability to utilize information resources and apply that ability to support lifelong learning. The language in this competency collapses information skills and behaviors into a single verb: “utilize.” The skills encompassed in “utilize” include all of the behaviors and skills necessary to generate and refine a successful search in an appropriate search engine, identify information that meets students' specific need, and synthesize that information to maintain the student's didactic body of knowledge.

The Pharm competency—“Maintain professional competence by identifying and evaluating emerging issues, trends, products, and services that may impact patient and population outcomes, management of resources and medication use systems, disease prevention services, and public health policy” [14]—explicitly states that the information skills and behaviors are identifying and evaluating multiple specific types of information. In this example, identifying the types of information to evaluate information indicates the program's emphasis is on students demonstrating the ability to evaluate information.

The way that these competencies focus on information skills and behaviors differs although both examples explicitly focus on information. The Medical competency is a broad statement that encompasses multiple elements of information use, such as knowing what places are appropriate for a student's specific question. In contrast, the Pharm example does not include the dimension of searching that includes knowing where to go to address a specific information need. Instead, the Pharm competency focuses on evaluating information, with a qualifying statement on identifying what information impacts their client population. Both competencies are about information skills and behaviors but highlight different dimensions.

### Implicit information skills and behaviors

**Figure 2:**
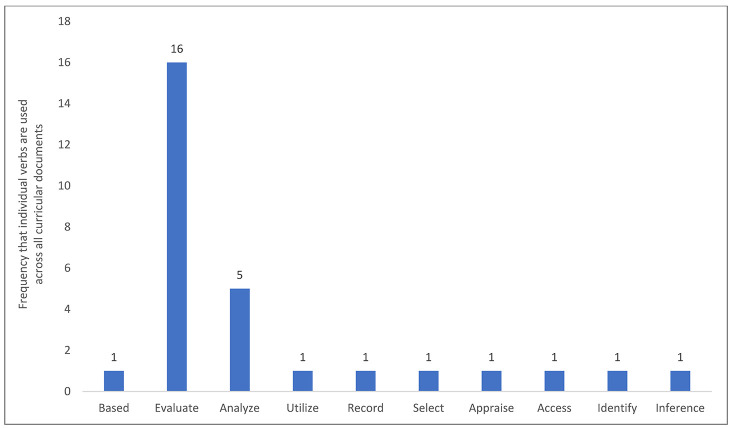
Analytic coding of action verbs used to describe information skills or behaviors in the professional program curricular competencies

Although there were few explicit statements about information skills or behaviors, the five programs' curricula had competencies that often implied information skills or behaviors (Figure 2).

Information skills and behaviors were described in a variety of ways depending on whether information skills or behaviors were described explicitly or implicitly and what the competency's category was. However, the most commonly used verb to describe information skills and behaviors was “evaluate.” Evaluation occurs during two of the stages of evidence-based medicine (EBM)/evidence-based veterinary medicine (EBVM) and lies at the intersection of at least two of the frames of the Association of College & Research Libraries (ACRL) Framework.

A specific PHEB competency requires students to “apply evidence-based approaches in the development and evaluation of social and behavioral science interventions” [15]. This competency's focus is the application of the evidence, which is the fourth step of EBP. However, before reaching the application step, a scenario must be developed and the knowledge gap articulated. Then, the information has to be identified, acquired, and appraised. Students need the requisite information skills and behaviors to demonstrate the competency. The prerequisite steps are implied because students must have demonstrated them to apply evidence-based approaches.

Similarly, a CVM curricular competency states that students should “evaluate risk factors for individual or groups of animals for medical conditions in common domestic species” [12]. Knowing these risk factors is didactic knowledge. However, medical knowledge is constantly being updated in a variety of ways. Therefore, the implication of this competency is that students will know the risk factors even if they change, which indicates an ongoing functional level of information skills and behaviors.

### Comparison of competencies in different curricula

The five programs' curricula are designed to train professionals for different careers and, therefore, have different goals. However, given the commonalities among health professions, some similarity in competencies could be expected.

The competencies are difficult to compare because different programs' sets of competencies were presented in different ways. However, comparing them establishes the groundwork for identifying similar library instructional opportunities among professional programs. Instructional resources can be developed strategically to serve several needs by identifying areas of similar library instruction.

One type of competency in the curricular documents included several main concepts that tied together to create a bigger picture. This collection of several main concepts, or clustered-concept competency, often contains multiple categories from the coding rubric. Pharm's curricular document contains ten competencies, each presenting a cluster of concepts. An example is: “Provide comprehensive patient-centered care by designing, implementing, evaluating, and continually refining pharmacy care plans based on best pharmacotherapy practices that incorporate health literacy cultural competence, and psychosocial and socioeconomic factors to optimize patient outcomes” [14].

An individual concept approach has a narrow scope, often focusing on one skill or task. PHEB had fifty-nine curricular competencies that were primarily written about individual concepts. A PHEB example of an individual concept approach is: “Explain unique circumstances related to public health in rural areas” [15].

**Figure 3:**
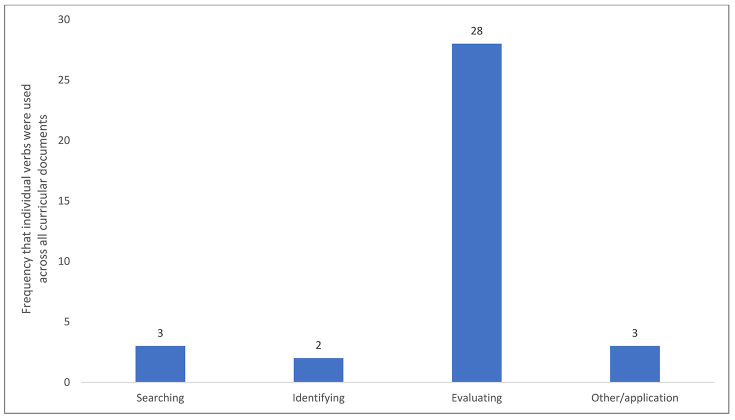
Categorizing analytic coding of action verbs

A similarity among the five programs' competencies is the frequent use of “evaluate” to both explicitly and implicitly describe information skills and behaviors (Figure 3). Other frequent verbs are categorized together by type. The evaluating category includes six separate verbs that are used in twenty-eight instances (Figure 3). The searching category includes two verbs that are used for a total of three instances. The identifying category includes one word used once. Two verbs were used in competencies that focused on other aspects so they are included in a category labeled other/application.

A total of thirty competencies had either implicit and explicit information skills and behaviors. These were categorized using the analytic coding of action verbs. There were five competencies that implied multiple types of information-searching behavior and skills. Each skill was represented separately, for a total of thirty-six coded action verbs. The searching category included the words “search” and “access.” The identifying category included only the verb “identify.” Six different verbs were included in the evaluating category: “interpret,” “analyze,” “evaluate,” “appraise,” “infer,” and “record” (due to the context of the competency's context). Two verbs did not fit in the other categories and were categorized in the other/application category: “develop” and “utilize.”

## DISCUSSION

This research explored what, if, and how information skills and behaviors were present in our stakeholders' curricular documents. Four of the five programs had competencies with explicitly stated information skills or behaviors. There were no more than two competencies in any individual professional program's curricular document that explicitly stated information skills or behaviors. However, all five programs had curricular goals that implied information skills or behaviors. This implicit requirement of information skills and behaviors among the professional programs' competencies highlights the need for developing a supporting instructional schema.

Differences in the curricular documents made comparing them challenging. For instance, some competencies were clustered-concept competencies, tying several types of skills together in a summative statement. These competencies were difficult to compare to individual-concept competencies. To generate a tangible way to compare curricular goals among the professional programs, a qualitative method was developed using sentence diagramming.

The curricular documents were condensed to the active verbs that were present in each competency that either explicitly or implicitly required demonstration of information skills or behaviors. This allowed comparison of the curricular documents for embedded information skills and behaviors. Of the eleven verbs used to describe information skills and behaviors, the single most common was “evaluate.” This commonality among the curricular documents indicates that a common goal among the five professional programs is their students' capability of evaluating information.

Evaluating information is central for many processes. It occurs during two steps of the EBP cycle: “Appraise” and “Assess” [2]. In the ACRL Framework, evaluating information falls at the intersection of at least two of the six frames: (1) authority is constructed and contextual, and (2) searching as strategic exploration [4]. Overlaying the methodology of EBP with the ACRL Framework highlighted the central role of information skills and behaviors. Applying the EBP/ACRL Framework–combined perspective with the curricular competencies of the stakeholders' programs may explain why there were so few explicit information skills and behaviors competencies; the information skills and behaviors were implicit to a professional career because an individual must be able to synthesize information for a given purpose [19].

Among the curricular documents, individual-concept competencies were about knowledge, comprehension of knowledge, or application of it, corresponding to the first three levels of Bloom's cognitive taxonomy [20]. In contrast, clustered-concept competencies incorporate multiple skills and processes. These clustered-concept competencies focused on higher-order processing such as analyzing, synthesizing, and evaluating information, falling within the higher-order levels of Bloom's cognitive taxonomy [20]. Competencies with the action verb “evaluate” required students to demonstrate higher-order thinking skills, including integrating knowledge for evaluation purposes.

Requiring students to arrive at evidence-based decisions through the synthesis of information is authentic to their future careers. Making this synthesis of information part of curricular goals—including the assessment—aligns classroom-based assessments with curricular documents [21]. Student learning may not include observable behaviors, making evaluating their learning complicated [22, 23]. Therefore, determining whether students have acquired foundational concepts such as information skills and behaviors can be difficult. Using objective structured clinical examinations (OSCEs) is one possible way to align assessments with competencies [24]. With this alignment, student performance becomes data that identify concepts that students are missing [25].

Teaching students transferable skills, such as information skills and behaviors, supports the professional curricula to transition students from the classroom to the workplace [26]. Therefore, library instruction can be scaffolded across professional programs that require similar information skills and behaviors, such as evaluating information. Deliberately scaffolding information skills and behaviors within the curriculum supports the entire learning process, from acquiring a foundational body of knowledge to applying concepts to new situations [27]. Identifying the individual instructional opportunities among the professional programs allows a longitudinal approach to library instructional support. This scaffolding of information skills and behaviors can guide students through experiential learning, specifically how to apply information in the context of their professional programs and future careers.

Creating an instruction schema to support stakeholders' educational goals involving information skills and behaviors is complex because their needs must first be identified. The differences and inconsistencies in the descriptions of information skills and behaviors illustrate the challenge in developing a sustainable and equitable instruction schema for the stakeholders' educational goals. In addition, not all the professional programs have mapped the curricula to their own written curricular competencies. Finally, individual instructors may have specific educational course goals that do not directly address a curricular competency. This occurs because curricular competencies encompass an entire curriculum that is composed of multiple courses. The complexity of supporting stakeholders' educational goals is further complicated by the unpublished educational goals of the administration, faculty, and accrediting boards. However, we have developed a method to identify information skills and behaviors in curricular documents that are not emphasizing, but requiring, these skills.

The diversity of inputs that must be included to create an MSL instruction schema that supports the curricula of multiple professional programs' curricular competencies is challenging for developing equitable support that is sustainable [28]. However, identifying the individual instructional opportunities among the professional programs allows a longitudinal approach to library instructional support.

**Figure 4:**
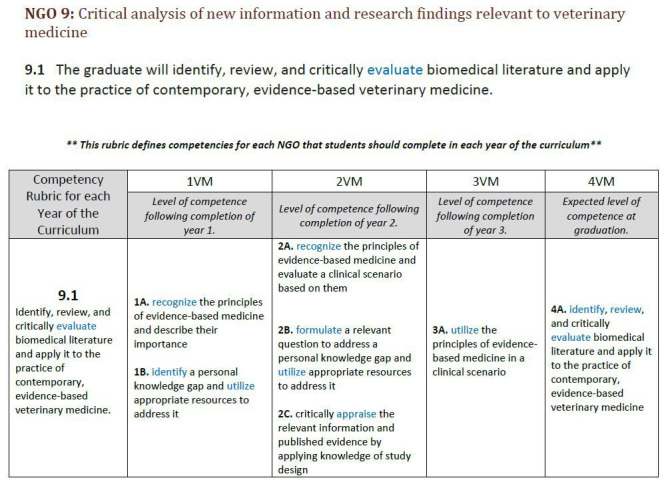
CVM's information skills and behaviors outcome

This study had several limitations. The five programs' curricular competency documents were collected in 2017, and several have changed. For example, the PHEB competencies were updated in 2018–2019. In addition, the curricular documents from the five programs are labeled differently: “New Graduate Outcomes & Competency Rubrics” (CVM) [12], “Competency Based Learning Objectives” (Medical) [11], “Graduate Student Learning Outcomes,” (Nursing) [13], “Professional Competencies” (Pharm) [14], and “Competencies” (PHEB) [15]. These titles suggest potential differences in thought about level of assessment for each program and reflects discrepancies in the education literature. One additional source of difficulty when comparing these documents was a difference in their level of detail. The CVM curricular document had overall stated outcomes and then benchmarks for students to demonstrate every year (Figure 4) [12]. We did not include this additional information in our analyses because none of the other programs had anything comparable. Lastly, we could not account for unstated objectives because we analyzed only the stated curricular documents.

CVM has mapped their curriculum to their curricular outcomes. This curricular outcome is explicitly about information skills and behaviors. There is an overall outcome and what the student achievement should be for every year they are in the program.

### Next steps

Having mined the five professional programs' curricular competency documents, we plan to contact the programs to align the data with the stakeholders' intentions. Doing so allows us to begin developing the MSL Instructional Data-Driven Schema (MIDDaS). This schema will provide both an intentional way to scaffold the skills and techniques that MSL offers to the professional programs. Matching this schema with the stakeholders' curricula will allow MSL to offer sustainable and equitable instruction.

## SUPPLEMENTAL FILES

**Appendix A:** Texas A&M University College of Medicine: Competency based learning objectives

**Appendix B:** Texas A&M University College of Nursing: Graduate student learning outcomes

**Appendix C:** Texas A&M University College of Veterinary Medicine & Biomedical Sciences: New graduate outcomes & competency rubrics

**Appendix D:** Texas A&M University Irma Lerma Rangel College of Pharmacy: Professional competencies

**Appendix E:** Texas A&M University School of Public Health: Competencies

**Appendix F:** Context of project and coding rubric
