# Supplementary material for: Identifying information literacy skills and behaviors in the curricular competencies of health professions
Source: J Med Libr Assoc. 2020 Jul 1;108(3):463–79. doi: 10.5195/jmla.2020.833 (PMC7441914; doi:10.5195/jmla.2020.833)
Supplement: Supplementary file 1 — Appendix A: Texas A&M University College of Medicine: Competency based learning objectives [file jmla-108-3-463-s01.pdf]

JMLA Journal of the Medical Library Association

SC

Supplemental content to
J Med Libr Assoc. Jul;108(3):dx.doi.org/10.5195/jmla.2020.833
www.jmla.mlanet.org
© Waltz, Moberly, Carrigan 2020

Identifying information literacy skills and behaviors in the curricular competencies of health professions

Micah J. Waltz; Heather K. Moberly, AHIP; Esther E. Carrigan, AHIP

APPENDIX A

Texas A&M University College of Medicine: Competency based learning objectives

TAMHSC DENTISTRY NURSING PHARMACY PUBLIC HEALTH Search

A|M MEDICINE TEXAS A&M UNIVERSITY

About Education Departments Administration Campuses Research & Graduate Studies Alumni Giving News

Competency Based Learning Objectives

Home » Curriculum Support » Competency Based Learning Objectives

Medical Knowledge

Medical students must demonstrate knowledge of established and evolving biomedical, clinical, and psychosocial sciences and their application to patient care.

Institutional Learning Objectives
Upon completion of the medical school curriculum, our students will be able to:

MK1	Demonstrate knowledge of normal human structure and function at the organ-system, tissue, cellular and molecular level; and of the interaction of human systems in maintaining homeostasis
MK2	Describe the basic mechanisms involved in the causation of human disease and their influence on clinical presentation and therapy
MK3	Demonstrate an understanding of how healthy lifestyles, psychosocial factors, and behavioral factors influence heath
MK4	Apply evidenced-based methods to clinical problem solving
MK5	Demonstrate an understanding of the epidemiology of common diseases within a population and the approaches which are useful in reducing their incidence and prevalence
MK6	Demonstrate knowledge of common societal problems such as domestic violence and substance abuse, including diagnosis, prevention, reporting, and treatment

Patient Care

Medical students must be able to provide appropriate, compassionate patient care for the promotion of health and treatment of health problems across the human life span.

Institutional Learning Objectives
Upon completion of the medical school curriculum, our students will be able to:

PC1	Obtain both complete and system-focused medical histories that include psychosocial and behavioral determinants of health
PC2	Perform both complete and system-focused physical examinations
PC3	Develop appropriate differential diagnoses by integrating collected clinical information
PC4	Develop contextual and individualized diagnostic and treatment plans based upon collected clinical information

Howdy · Directory · Maps · Jobs · Email · IT

Curriculum Support

- Information for Visiting Students
- › *Competency Based Learning Objectives*
- Patient Encounter and Procedure Information +
- Course Creation Information
- Student Forms +
- Rotations +
- Student Initiated Electives +
- Objectively Structured Clinical Examination (OSCE) +
- Curriculum Overview
- Foundations and Organ Systems

PC5	Interpret the results of commonly used laboratory and radiologic studies
PC6	Recognize common immediately life-threatening conditions and initiate therapy
PC7	Formulate an initial management plan for critically ill patients
PC8	Demonstrate an understanding of the principles involved in the care of patients across the spectrum of the human life cycle
PC9	Perform technical procedures including: venipuncture and arterial puncture; insertion of intravenous, central venous, and urethral catheters; insertion of a nasogastric tube; lumbar puncture; basic suturing; and basic airway management
PC10	Discuss the principles of pain management and formulate a basic multidisciplinary care plan
PC11	Assist in the provision of appropriate end-of-life care within a multidisciplinary team
PC12	Educate patients in personalized health maintenance
PC13	Perform basic health risk assessment and formulate appropriate screening plans
PC14	Properly utilize clinical, laboratory, radiologic, and pathologic examinations to diagnose and treat common maladies
PC15	Formulate preventive, curative, rehabilitative, and palliative therapeutic strategies for common disorders

Interpersonal and Communication Skills

Medical students must demonstrate interpersonal and communication skills that result in the effective and respectful exchange of information and collaboration with patients, patients' family members, and health professionals across a broad range of socioeconomic and cultural backgrounds.

Institutional Learning Objectives

Upon completion of the medical school curriculum, our students will be able to:

ICS1	Demonstrate effective listening skills
ICS2	Discuss diagnostic and treatment options in a manner comprehensible to the patient
ICS3	Communicate effectively with patients, patients' family members, peers, and other members of the health care team
ICS4	Educate patients, patients' family members, peers, and other members of the health care team at an appropriate level using appropriate technologies
ICS5	Maintain accurate medical records

Professionalism

Medical students must demonstrate a commitment to professional responsibilities, adherence to ethical behaviors, and sensitivity to a diverse patient population.

Institutional Learning Objectives

Upon completion of the medical school curriculum, our students will be able to:

PROF1	Demonstrate an understanding of legal and ethical principles governing the physician-patient relationship
PROF2	Display honesty, integrity and ethical behavior

PROF3	Act in the patient's best interest and serve as a patient advocate
PROF4	Treat patients and patients' family members respectfully and compassionately, regardless of age, disability, gender, race, ethnicity, culture, religion, sexual preference, and socio-economic status
PROF5	Respect the privacy of patients
PROF6	Work with other health professionals in a collaborative fashion
PROF7	Demonstrate an awareness of leadership roles in medicine and society
PROF8	Recognize potential conflicts of interest and demonstrate awareness of appropriate courses of action
PROF9	Demonstrate an understanding of peer review and the expectations of professional licensing boards, including medical jurisprudence
PROF10	Demonstrate knowledge of responsibilities to patients, peers, and other members of the health care team
PROF11	Respond to conflicts in a professional manner
PROF12	Project a professional image in demeanor and personal appearance

Systems-Based Practice

Medical students must demonstrate an awareness of the larger context and system of health care and their impact on patient care.

Institutional Learning Objectives

Upon completion of the medical school curriculum, our students will be able to:

SBP1	Apply knowledge of health care systems to improve and optimize patient care
SBP2	Advocate for continuous quality improvement in patient care and patient safety
SBP3	Demonstrate an understanding of cost containment principles and their application in the delivery of health care
SBP4	Demonstrate an understanding of the legal and regulatory frameworks governing the practice of medicine which affect payment, reimbursement, referrals and incentives
SBP5	Recognize various approaches to the organization, financing, and delivery of health care
SBP6	Utilize information technology in providing medical care for individuals
SBP7	Recognize health care system deficiencies regarding social needs, access to care issues, and health disparities when they arise and develop strategies for optimal care of each individual patient.

Practice-Based Learning and Improvement

Medical students must demonstrate the ability to investigate and evaluate their care of patients, to appraise and assimilate scientific evidence, and to continuously improve their patient care skills based on self-evaluation and life-long learning.

Institutional Learning Objectives

Upon completion of the medical school curriculum, our students will be able to:

PBLI1	Identify and rectify deficiencies in their knowledge base and skill set
PBLI2	Incorporate formative feedback into personal performance
PBLI3	Accomplish learning and improvement goals with appropriate self-directed activities
PBLI4	Utilize information resources and available data to support life-long learning
PBLI5	Select, appraise, and utilize evidence from scientific studies related to clinical questions and patients' health problems
PBLI6	Demonstrate an understanding of the basic principles and importance of scholarly activity and Translational Research in the practice of medicine

Cultural Competence

Medical students should have the capacity to function effectively as a individual within the context of the cultural beliefs, practices, and needs presented by patients and their communities.

Institutional Learning Objectives

Upon completion of the medical school curriculum, our students will be able to:

CC1	Demonstrate an understanding of the manner in which diverse cultures and belief systems perceive health and illness and respond to various symptoms, diseases, and treatments.
CC2	Recognize and appropriately address gender and cultural biases in themselves, in others, and in the process of health care delivery.

Texas A&M College of Medicine
3050 Health Professions Education Building
8447 Riverside Pkwy
Bryan, TX 77807
979-436-0200 | 979-436-0092
