# Supplementary material for: Identifying information literacy skills and behaviors in the curricular competencies of health professions
Source: J Med Libr Assoc. 2020 Jul 1;108(3):463–79. doi: 10.5195/jmla.2020.833 (PMC7441914; doi:10.5195/jmla.2020.833)
Supplement: Supplementary file 2 — Appendix B: Texas A&M University College of Nursing: Graduate student learning outcomes [file jmla-108-3-463-s02.pdf]

Journal of the Medical Library Association

Supplemental content to
J Med Libr Assoc. Jul;108(3):dx.doi.org/10.5195/jmla.2020.833
www.jmla.mlanet.org
© Waltz, Moberly, Carrigan 2020

Identifying information literacy skills and behaviors in the curricular competencies of health professions

Micah J. Waltz; Heather K. Moberly, AHIP; Esther E. Carrigan, AHIP

APPENDIX B

Texas A&M University College of Nursing: Graduate student learning outcomes

TAMHSC DENTISTRY MEDICINE PHARMACY PUBLIC HEALTH Search

NURSING
TEXAS A&M UNIVERSITY

About Contact Future Students Current Students Alumni Faculty & Staff Administration Research Giving

Graduate Student Learning Outcomes

Home » Future » Graduate Student Learning Outcomes

Howdy · Directory · Maps · Jobs · Email · IT

Future Students

- Additional Program Information
- Essential Competency Requirements
- Criminal Background Check
- Excess Credit Hour Guideline
- Laptop Requirement
- Admissions Assessment Exam
- International Applicants
- Baccalaureate Student Learning Outcomes
- FAQs
- › *Graduate Student Learning Outcomes*

Expected Student Outcomes: MSN-Education

At the completion of the MSN, graduates of the College of Nursing will be prepared to:

1. Integrate scientific findings from nursing, biophysical science, genetics, public health, quality improvement and organizational sciences for the continual improvement of nursing care across diverse settings.
2. Utilize organizational and systems leadership in developing working relationships and making ethical and critical decisions to promote quality and safe care.
3. Apply principles, methods and tools of quality improvement within an organization to promote quality care.
4. Work as a change agent to apply and disseminate research outcomes within the practice setting.
5. Use patient care and communication technologies to enhance patient care.
6. Intervene at the system level to develop and implement policies that influence health care.
7. Collaborate with other health care professionals to manage and coordinate care.
8. Integrate organizational, client centered and culturally centered approaches to plan, deliver and evaluate health care for individuals, families and populations.
9. Influence health care outcomes by integrating advanced knowledge into direct and indirect care.

Expected Student Outcomes: MSN-Family Nurse Practitioner

At the completion of the MSN, graduates of the College of Nursing will be prepared to:

1. Conduct a comprehensive and systematic assessment of health and illness incorporating ethical, diverse and culturally sensitive approaches.
2. Design, implement and evaluate therapeutic and preventative interventions based on nursing science, and other sciences and humanities.
3. Develop and sustain therapeutic relationships and partnerships with individuals, families or populations.
4. [illegible]

evidence-based care to improve patient outcomes.

6. Demonstrate organizational and systems leadership in the provision of quality and safe patient care.
7. Integrate principles of quality improvement, informatics and current and emerging technologies to optimize patient outcomes.
8. Guide, mentor and support nurses and others to achieve excellence in patient care.
9. Educate and guide individuals and groups in health-related transitions throughout the lifespan.
10. Analyze the links among practice, organizational, population, fiscal and policy issues to advocate for improved patient outcomes.

MEMBER OF THE TEXAS A&M UNIVERSITY SYSTEM

NLN National League for Nursing Center of Excellence in Nursing Education

AHC

CCNE ACCREDITED

The baccalaureate and the master's degrees in nursing at the Texas A&M University College of Nursing are accredited by the Commission on Collegiate Nursing Education.
