# Supplementary material for: Identifying information literacy skills and behaviors in the curricular competencies of health professions
Source: J Med Libr Assoc. 2020 Jul 1;108(3):463–79. doi: 10.5195/jmla.2020.833 (PMC7441914; doi:10.5195/jmla.2020.833)
Supplement: Supplementary file 3 — Appendix C: Texas A&M University College of Veterinary Medicine & Biomedical Sciences: New graduate outcomes & competency rubrics [file jmla-108-3-463-s03.pdf]

JMLA Journal of the Medical Library Association

Supplemental content to
J Med Libr Assoc. Jul;108(3):dx.doi.org/10.5195/jmla.2020.833
www.jmla.mlanet.org
© Waltz, Moberly, Carrigan 2020

Identifying information literacy skills and behaviors in the curricular competencies of health professions

Micah J. Waltz; Heather K. Moberly, AHIP; Esther E. Carrigan, AHIP

APPENDIX C

Texas A&M University College of Veterinary Medicine & Biomedical Sciences: New graduate outcomes & competency rubrics

New Graduate Outcomes & Competency Rubrics

VETERINARY MEDICINE & BIOMEDICAL SCIENCES
TEXAS A&M UNIVERSITY

NGO 1:
Comprehensive patient diagnosis (problem-solving skills), appropriate use of clinical laboratory testing, and record management

NGO 2:
Comprehensive treatment planning, including patient referral when indicated

NGO 3:
Anesthesia and pain management, patient welfare

NGO 4:
Basic surgery skills, experience, and case management

NGO 5:
Basic medicine skills, experience, and case management

NGO 6:
Emergency and intensive care case management

NGO 7:
Health promotion, disease prevention/biosecurity, zoonosis, and food safety

NGO 8:
Client communications and ethical conduct

NGO 9:
Critical analysis of new information and research findings relevant to veterinary medicine

NGO 10:
Team collaboration, leadership, and practice management

NGO 11:
Multicultural awareness and personal wellness

NGO 12:
Legal and regulatory compliance

NGO 13:
Animal welfare

NGO 1: Comprehensive patient diagnosis (problem-solving skills), appropriate use of clinical laboratory testing, and record management

1.1 The graduate will obtain patient signalment, history, and physical examination and formulate a problem list and differential diagnoses based upon their findings for individual or groups of common domestic species.

***** This rubric defines competencies for each NGO that students should complete in each year of the curriculum*****

Competency Rubric for each Year of the Curriculum	1VM	2VM	3VM	4VM
	Level of competence following completion of year 1.	*Level of competence following completion of year 2.*	*Level of competence following completion of year 3.*	*Expected level of competence at graduation.*
**1.1**<br>Obtain patient signalment, history, and physical examination and formulate a problem list and differential diagnoses based upon their findings for individual or groups of common domestic species.	**1A.** define signalment and obtain a complete history for common domestic species<br><br>**1B.** perform complete physical examination of all body systems on common domestic species<br><br>**1C.** obtain history for a herd/group environment<br><br>**1D.** differentiate normal from abnormal patient findings<br><br>**1E.** develop a problem list using appropriate medical terminology based on signalment, history and physical exam	**2A.** interpret focused history to identify body system(s) involved etiopathogenesis<br><br>**2B.** differentiate normal from abnormal physical exam parameters<br><br>**2C.** perform physical examination on core species in clinical scenario<br><br>**2D.** develop a problem list using appropriate medical terminology based on signalment, history and physical exam scenarios<br><br>**2E.** create a differential diagnosis list at the level of body system	**3A.** obtain patient signalment, history, and perform and interpret physical examination in a clinical scenario<br><br>**3B.** formulate a prioritized problem list and differential diagnoses based upon their findings in a clinical scenario	**4A.** Obtain patient signalment, history, and perform and interpret a physical examination in a clinical patient<br><br>**4B.** formulate a prioritized problem list and differential diagnoses based upon their findings for clinical patients

Examples of session-level/meeting-level/unit-level outcomes to use as a guide to fulfill **NGO 1.1**:

- use knowledge of anatomy and physiology to perform a complete systematic physical examination of all body systems, distinguishing normal and abnormal findings,
- obtain a signalment and complete history to collect relevant information about the patient(s),
- develop an accurate and complete problem list, based on the history and physical examination, and use semantic qualifiers to develop a concise patient problem statement,
- list and describe the common and significant diseases that are developmental, degenerative, metabolic, nutritional, neoplastic, infectious, immune mediated, toxic, and traumatic in nature for each of the common domestic species,
- develop a preliminary differential diagnosis list, based on the history and physical examination of the patient(s), apply clinical reasoning skills to rank the possible medical conditions from most likely to least likely.

CC BY-NC This work is licensed under a Creative Commons Attribution-NonCommercial 4.0 International

1.2 The graduate will construct an appropriate diagnostic plan, based upon their preliminary differential diagnosis list, and analyze the findings in order to reach a presumptive diagnosis of common medical conditions in common domestic species.

***** This rubric defines competencies for each NGO that students should complete in each year of the curriculum*****

Competency Rubric for each Year of the Curriculum	1VM	2VM	3VM	4VM
	Level of competence following completion of year 1.	*Level of competence following completion of year 2.*	*Level of competence following completion of year 3.*	*Expected level of competence at graduation.*
**1.2**<br>Construct an appropriate diagnostic plan, based upon their preliminary differential diagnosis list, and analyze the findings in order to reach a presumptive diagnosis of common medical conditions in common domestic species.	**1A.** match core diagnostic tests to appropriate body system(s) and appropriate sample<br><br>**1B.** perform core diagnostic tests and interpret test results	**2A.** match available diagnostic tests to differential diagnosis list<br><br>**2B.** prioritize available diagnostic tests based on their benefits and risks<br><br>**2C.** perform and interpret core diagnostic tests<br><br>**2D.** communicate diagnostic plan with associated risks to stakeholders in a clinical scenario	**3A.** construct an appropriate diagnostic plan(s), based upon their preliminary differential diagnosis list in a clinical scenario<br><br>**3B.** analyze diagnostic findings in order to reach a presumptive diagnosis of common medical conditions in a clinical scenario<br><br>**3C.** evaluate available diagnostic tests and identify benefits, risks, costs and client constraints in a clinical scenario<br><br>**3D.** communicate diagnostic plan with stakeholders in a clinical scenario	**4A.** construct an appropriate diagnostic plan, based upon their preliminary differential diagnosis list and client constraints for a clinical patient<br><br>**4B.** analyze diagnostic findings in order to reach a presumptive diagnosis of common medical conditions in clinical patients<br><br>**4C.** evaluate available diagnostic tests and identify benefits, risks, costs and client/patient constraints in a clinical patient<br><br>**4D.** communicate diagnostic plan with stakeholders on clinical patients

Examples of session-level/meeting-level/unit-level outcomes to use as a guide to fulfill **NGO 1.2**:

- explain the pathophysiology that may occur in the major organ systems for each of the common and significant diseases,
- identify appropriate diagnostic tests and procedures in order to rule in or rule out medical conditions based on the preliminary differential diagnosis list,
- weigh the potential benefits and risks of performing diagnostic tests on the patient, as well as recognizing any client financial constraints, in order to develop a definitive diagnostic plan,
- perform diagnostic tests and procedures, interpret the laboratory data and other test results, and accurately apply this information in order to determine an accurate diagnosis for common medical conditions.

CC BY-NC This work is licensed under a Creative Commons Attribution-NonCommercial 4.0 International

1.3 The graduate will prepare a medical record, documenting all relevant client and patient information, and communicate effectively with the animal health care team using the medical record.

***** This rubric defines competencies for each NGO that students should complete in each year of the curriculum*****

Competency Rubric for each Year of the Curriculum	1VM	2VM	3VM	4VM
	Level of competence following completion of year 1.	*Level of competence following completion of year 2.*	*Level of competence following completion of year 3.*	*Expected level of competence at graduation.*
**1.3**<br>Prepare a medical record, documenting all relevant client and patient information, and communicate with the animal health care team using the medical record.	**1A.** define the components of a complete medical record using a standardized SOAP format<br><br>**1B.** prepare a medical record (SO)<br><br>**1C.** explain the legal and regulatory aspects of a medical record	**2A.** evaluate and assess a medical record for completeness and legal compliance<br><br>**2B.** prepare a medical record (SOAP)	**3A.** create a medical record (SOAP) that complies with legal requirements in a clinical scenario<br><br>**3B.** communicate with the animal health care team using the medical record in a clinical scenario	**4A.** prepare a medical record (SOAP), documenting all relevant client and patient information<br><br>**4B.** communicate with the animal health care team using the medical record for a clinical patient

Examples of session-level/meeting-level/unit-level outcomes to use as a guide to fulfill **NGO 1.3**:

- prepare a medical record that concisely summarizes all relevant patient information, utilizing a standard veterinary medical format that is readily understood by the health care team,
- prepare a medical record that is legible, accurate, current, and conforms to all legal requirements.

This work is licensed under a Creative Commons Attribution-NonCommercial 4.0 International

NGO 2: Comprehensive treatment planning, including patient referral when indicated

2.1 The graduate will formulate a comprehensive therapeutic plan for medical and surgical problems based upon their diagnosis for common domestic species.

***** This rubric defines competencies for each NGO that students should complete in each year of the curriculum*****

Competency Rubric for each Year of the Curriculum	1VM	2VM	3VM	4VM
	Level of competence following completion of year 1.	*Level of competence following completion of year 2.*	*Level of competence following completion of year 3.*	*Expected level of competence at graduation.*
**2.1** Formulate a comprehensive therapeutic plan for medical and surgical problems based upon their diagnosis for common domestic species.	**1A**. identify the therapeutic plan as a component of SOAP	**2A.** investigate and evaluate available therapeutic options for a given disease/diagnosis in terms of potential benefits and risks<br><br>**2B.** evaluate pre-formulated treatment plan in a clinical scenario	**3A.** formulate multiple therapeutic plans for a clinical scenario, accounting for patient and client constraints<br><br>**3B.** formulate and communicate treatment plans in a clinical scenario	**4A.** formulate comprehensive therapeutic plan including follow-up for medical and surgical problems in a clinical patient, accounting for patient and client constraints<br><br>**4B.** communicate treatment options for acute and follow-up care with client and healthcare team for a clinical patient

Examples of session-level/meeting-level/unit-level outcomes to use as a guide to fulfill **NGO 2.1**:

- list a range of potential therapeutic options for a given diagnosis,
- weigh the potential benefits and risks of the available therapeutic options and consider the feasibility of implementation, owner compliance, and the financial constraints of the client for a given patient,
- formulate a comprehensive therapeutic plan for medical and surgical problems based upon the diagnosis for common domestic species,
- communicate the therapeutic options to the client in a manner that is readily understood,
- promotes adherence to recommendations, and facilitates the selection of a treatment option.

CC BY-NC This work is licensed under a Creative Commons Attribution-NonCommercial 4.0 International

2.2 Evaluate the scope of their personal and professional limits and appropriately judge when to seek professional advice, assistance, and support and manage a referral case.

***** This rubric defines competencies for each NGO that students should complete in each year of the curriculum*****

Competency Rubric for each Year of the Curriculum	1VM	2VM	3VM	4VM
	Level of competence following completion of year 1.	*Level of competence following completion of year 2.*	*Level of competence following completion of year 3.*	*Expected level of competence at graduation.*
**2.2**<br>Evaluate the scope of their personal and professional limits and appropriately judge when to seek professional advice, assistance, and support and manage a referral case.	**1A**. apply appropriate terminology when communicating with stakeholders<br><br>**1B.** contrast communication needs between DVM and Client<br><br>**1C.** match and apply terminology used in communication with different audience (ie, specialist DVM vs owner)	**2A.** differentiate roles of general and referral practices in veterinary medicine<br><br>**2B.** list and explain requirements (equipment, cost, expertise, staff) necessary to perform a given diagnostic test or procedure<br><br>**2C.** explain referral process to a client in a clinical scenario	**3A.** identify personal and professional strengths and limits as components of case management<br><br>**3B.** identify appropriate clinical scenarios in which to refer a patient<br><br>**3C**. communicate referral process, costs and expectations with the client in a clinical scenario<br><br>**3D.** communicate with referral practice personnel in a clinical scenario	**4A.** differentiate clinical cases which require referral from general care in a clinical patient<br><br>**4B.** manage referral process and communicate with stakeholders throughout all stages

Examples of session-level/meeting-level/unit-level outcomes to use as a guide to fulfill **NGO 2.2**:

- describe the roles and responsibilities of referring and referral veterinarians and explain the importance of referral in providing optimal patient care,
- judge their level of expertise and discriminate between patients they are capable of treating and patients that require referral or other forms of assistance,
- state the requirements necessary to perform a given diagnostic test or procedure, including staff, equipment, expertise, and other relevant considerations,
- communicate with the owner and the referral veterinarian to coordinate patient care before, during, and after the referral, including preparing the medical records,
- explain the referral process, including selecting a referral DVM with appropriate expertise, preparing medical records, communicating with the referral veterinarian, and communicating with the owner, including providing recommendations for long-term care and recheck appointments.

(cc) BY-NC This work is licensed under a Creative Commons Attribution-NonCommercial 4.0 International

NGO 3: Anesthesia and pain management, patient welfare

3.1 The graduate will recognize the need for sedation and anesthesia and evaluate the pertinent patient information in order to formulate an appropriate anesthesia protocol in common domestic species.

***** This rubric defines competencies for each NGO that students should complete in each year of the curriculum*****

Competency Rubric for each Year of the Curriculum	1VM	2VM	3VM	4VM
	Level of competence following completion of year 1.	*Level of competence following completion of year 2.*	*Level of competence following completion of year 3.*	*Expected level of competence at graduation.*
**3.1** Recognize the need for sedation, anesthesia and analgesia and evaluate the pertinent patient information in order to formulate an appropriate anesthesia protocol in common domestic species.	**1A.** identify pathways of pain<br><br>**1B.** recognize legal and ethical responsibilities of sedation/anesthesia/analgesia<br><br>**1C.** identify the responsibility of the veterinarian to alleviate pain and suffering	**2A.** recognize causes of pain and the need for sedation/anesthesia/analgesia<br><br>**2B.** evaluate healthy patient prior to sedation/anesthesia/analgesia in a clinical scenario<br><br>**2C.** formulate and justify sedation/anesthesia/analgesia protocol for a healthy patient in a clinical scenario<br><br>**2D.** describe considerations for co-morbidities on sedation/anesthesia/analgesia protocols in a clinical scenario	**3A.** recognize pain in a patient in a clinical scenario and formulate a treatment plan<br><br>**3B.** evaluate patient prior to sedation/anesthesia/analgesia in a clinical scenario<br><br>**3C.** formulate and justify sedation/anesthesia protocol for healthy patients and those with co-morbidities in a clinical scenario	**4A.** recognize pain and implement a treatment plan to address pain in a clinical patient<br><br>**4B.** evaluate clinical patient prior to sedation/anesthesia/analgesia<br><br>**4C.** formulate and justify sedation/anesthesia/analgesia protocol for a clinical patient, accounting for patient and owner constraints

Examples of session-level/meeting-level/unit-level outcomes to use as a guide to fulfill **NGO 3.1**:

- use knowledge of anatomy and physiology to evaluate the patient prior to formulating an anesthetic protocol,
- recognize clinical procedures likely to induce pain and recognize the responsibility of veterinarians to alleviate animal pain and suffering,
- state the indications and limitations of local and regional anesthetic techniques, short-and long-term injectable and inhalant anesthetic agents,
- classify common anesthetic and perianesthetic agents and explain their mechanisms of action, pharmacologic properties, and physiologic effects on the patient,
- evaluate pertinent patient information to formulate a safe and effective preanesthetic and anesthetic protocol for common procedures based on patient signalment, clinical examination findings, patient risk factors, financial considerations of the owner, and feasibility (e.g., equipment, personnel, competence),
- predict the expected degree of pain and employ an appropriate anesthetic protocol in order to adhere to appropriate standards of patient care with regard to the prevention and alleviation of pain.

This work is licensed under a Creative Commons Attribution-NonCommercial 4.0 International

3.2 The graduate will apply an appropriate anesthesia protocol to induce, maintain, and recover patients from anesthesia following current veterinary medical standards for common domestic species.

***** This rubric defines competencies for each NGO that students should complete in each year of the curriculum*****

Competency Rubric for each Year of the Curriculum	1VM	2VM	3VM	4VM
	Level of competence following completion of year 1.	*Level of competence following completion of year 2.*	*Level of competence following completion of year 3.*	*Expected level of competence at graduation.*
**3.2**<br>Apply an appropriate anesthesia protocol to induce, maintain, and recover patients from anesthesia following current veterinary medical standards for common domestic species.	**1A.** describe ventilation and circulation in a healthy patient<br><br>**1B.** describe anatomy relevant to performing local and regional anesthetic blocks	**2A.** identify benefits and risks of anesthesia in a clinical scenario<br><br>**2B.** apply anesthetic protocol in a clinical scenario<br><br>**2C.** identify the components and legal requirements of an anesthetic record	**3A.** apply anesthetic protocol and maintain as needed in a clinical scenario<br><br>**3B.** create complete anesthetic record	**4A.** implement an appropriate anesthesia protocol to induce, maintain, and recover patients from anesthesia in a clinical patient

Examples of session-level/meeting-level/unit-level outcomes to use as a guide to fulfill **NGO 3.2**:

- locate and describe the regional anatomy relevant to performing local and regional anesthetic techniques,
- describe the normal physiologic processes relevant to monitoring a patient during the anesthetic period,
- recognize the important legal and ethical responsibilities of veterinarians and their support staff with respect to roles and responsibilities during the induction, maintenance, and recovery of patients from anesthesia,
- name anesthetic equipment and monitoring devices that are commonly used during the induction, maintenance, and recovery of patients from anesthesia and explain the indications and limitations of each,
- choose and operate appropriate anesthetic equipment and monitoring devices that are commonly used to induce, maintain, and recover patients from anesthesia,
- perform local and regional anesthetic techniques and administer sedation and short- and long-term injectable and inhalant anesthetic agents safely and effectively,
- name common surgical procedures that can be performed under field conditions and explain the benefits, limitations, and risks of field anesthesia,
- monitor patients during the anesthetic period (i.e., before, during, and after surgery) and adjust and modify anesthetic protocols as needed to appropriately manage the patient,
- identify risks posed to patients and their handlers during the anesthetic period (i.e., before, during, and after surgery) and employ appropriate actions to reduce these risks and minimize potential patient complications.

CC BY-NC This work is licensed under a Creative Commons Attribution-NonCommercial 4.0 International

3.3 The graduate will recognize the clinical signs associated with pain in all common domestic species, assess the type of pain, classify the level of pain, formulate a pain management plan, and apply a therapy to effectively prevent and alleviate animal suffering and pain.

***** This rubric defines competencies for each NGO that students should complete in each year of the curriculum*****

Competency Rubric for each Year of the Curriculum	1VM	2VM	3VM	4VM
	Level of competence following completion of year 1.	*Level of competence following completion of year 2.*	*Level of competence following completion of year 3.*	*Expected level of competence at graduation.*
**3.3**<br>Recognize the clinical signs associated with pain in all common domestic species, assess the type of pain, classify the level of pain, formulate a pain management plan, and apply a therapy to prevent and alleviate animal suffering and pain.	**1A.** describe pathophysiology of pain<br><br>**1B.** recognize the clinical signs of pain on a physical exam<br><br>**1C.** describe veterinary pain scales	**2A.** apply pain scales in a clinical scenario<br><br>**2B.** formulate and justify pain management plan in a clinical scenario<br><br>**2C.** communicate a pain management plan to client and healthcare team in a clinical scenario	**3A.** formulate and justify pain management plan in a clinical scenario, accounting for client and patient constraints<br><br>**3B.** implement pain management plan in a clinical scenario	**4A.** apply pain scales in a clinical patient<br><br>**4B.** formulate and justify pain management plan in a clinical patient, accounting for client and patient constraints<br><br>**4C.** implement pain management plan in a clinical patient<br><br>**4D.** communicate pain management plan to client and healthcare team for a clinical patient

Examples of session-level/meeting-level/unit-level outcomes to use as a guide to fulfill **NGO 3.3**:

- explain the physiology of pain and apply knowledge of anatomy to recognize the clinical signs associated with pain in all common domestic species,
- explain the role of pain recognition scales in evaluating patients for pain and be able to apply those pain scales to common domestic species,
- recognize the need for pharmacologic, multimodal, and alternative therapies to address patient pain,
- list the classes of drugs that can be used to control pain, describe their mechanism of action, and describe the adverse reactions that may occur as a result of these actions,
- construct a pain management plan that matches the type and level of pain being experienced by the patient with the appropriate class, dose, and route of administration of drug to effectively alleviate the pain,
- weigh the benefits of pain management against the potential complications of pharmaceutical intervention when developing a pain management plan,
- implement a comprehensive pain management plan and communicate long-term monitoring and treatment schemes with members of the health care team and owners.

(cc) BY-NC This work is licensed under a Creative Commons Attribution-NonCommercial 4.0 International

NGO 4: Basic surgery skills, experience, and case management

4.1 The graduate will safely, consistently, and effectively perform the core surgical skills required to successfully carry out any entry-level surgical procedure in common domestic species.

***** This rubric defines competencies for each NGO that students should complete in each year of the curriculum*****

Competency Rubric for each Year of the Curriculum	1VM	2VM	3VM	4VM
	Level of competence following completion of year 1.	*Level of competence following completion of year 2.*	*Level of competence following completion of year 3.*	*Expected level of competence at graduation.*
**4.1**<br>Perform the core surgical skills required to carry out any entry-level surgical procedure in common domestic species.	**1A.** explain anatomical relationships associated with entry-level surgical procedures<br><br>**1B.** explain the physiological considerations for entry-level surgical procedures<br><br>**1C.** identify and safely handle instruments required to perform entry-level surgical procedures in common domestic species	**2A.** match instrument(s) with correct entry-level surgical procedures in common domestic species<br><br>**2B.** match tissue layer with appropriate suture material and suture pattern used in entry-level surgical procedures in common domestic species<br><br>**2C.** perform aseptic technique<br><br>**2D.** perform the core surgical skills required to carry out selected entry-level surgical procedures in common domestic species in a clinical scenario	**3A.** perform the core surgical skills required to carry out entry-level surgical procedures in common domestic species in a clinical scenario	**4A.** perform the core surgical skills required to carry out any entry-level surgical procedure in common domestic species in a clinical patient

Examples of session-level/meeting-level/unit-level outcomes to use as a guide to fulfill **NGO 4.1**:

- locate and describe the regional anatomy relevant to performing entry-level surgical procedures, including surgical landmarks and approaches,
- distinguish surgical procedures that require strict aseptic technique versus those that can be performed in the field, perform surgical scrubbing, gowning, and gloving in order to perform aseptic surgery,
- prepare a surgical site for aseptic surgery, apply the proper technique to maintain sterility during surgery, and recognize when sterility is broken during surgery,
- identify the major classes and common types of surgical instruments and state their appropriate surgical applications,
- demonstrate the proper grips and handling of each of the five basic classes of surgical instruments (i.e., scalpels, needle holders, clamps, scissors, and forceps),
- identify anatomical tissue layers and explain important considerations when handling them during surgery, including making and closing incisions,
- perform tissue handling techniques correctly during surgery using appropriate instruments to minimize tissue trauma,
- identify the major classes of suture material and describe their appropriate surgical applications,
- select the appropriate suture pattern for the surgical situation and state the advantages and disadvantages of each,
- perform the correct technique to tie surgical knots, explain their indications, and recognize when an improper ligature or pattern has been placed,
- predict potential complications of surgery and respond appropriately to complications when they arise,
- identify common types of drains, state their appropriate applications, and demonstrate the proper techniques to place them,
- identify common types of bandages, state their appropriate applications, and demonstrate the proper techniques to place them, as well as describe the potential complications when placed incorrectly.

(cc) BY-NC This work is licensed under a Creative Commons Attribution-NonCommercial 4.0 International

4.2 The graduate will select appropriate surgical interventions, based upon the identification of pathologic conditions and the evaluation of patient conditions, to treat surgical problems in common domestic species.

***** This rubric defines competencies for each NGO that students should complete in each year of the curriculum*****

Competency Rubric for each Year of the Curriculum	1VM	2VM	3VM	4VM
	Level of competence following completion of year 1.	*Level of competence following completion of year 2.*	*Level of competence following completion of year 3.*	*Expected level of competence at graduation.*
**4.2**<br>Select appropriate surgical interventions, based upon the identification of pathologic conditions and the evaluation of patient conditions, to treat surgical problems in common domestic species.	**1A.** describe normal physiologic conditions and anatomical presentations relevant to surgical intervention	**2A.** identify pathologic conditions and abnormal anatomical presentations that may be managed with surgical intervention	**3A.** select appropriate surgical interventions, based upon the identification of pathologic conditions and the evaluation of patient conditions, to treat surgical problems in common domestic species in a clinical scenario<br><br>**3B.** formulate and communicate the benefits, risks, and costs of a surgical procedure in a clinical scenario	**4A.** select appropriate surgical interventions, based upon the identification of pathologic conditions and the evaluation of patient conditions, to treat surgical problems in common domestic species in a clinical patient<br><br>**4B.** formulate and communicate the benefits, risks, and costs of a surgical procedure in a clinical patient

Examples of session-level/meeting-level/unit-level outcomes to use as a guide to fulfill **NGO 4.2**:

- identify normal anatomical structures and recognize altered anatomical relationships between structures on physical exam and/or specialized exam (i.e., lameness exam, ophthalmic exam) or via imaging modalities and describe what structures are altered and/or damaged,
- recognize the need for surgical intervention based upon interpretation of clinical signs and diagnostic tests,
- use knowledge of physiology to evaluate the physical status of the patient and identify abnormalities that should be addressed prior to surgery,
- describe surgical procedures that are appropriate for a given patient and explain the advantages and disadvantages of each procedure,
- formulate a surgical treatment plan for a patient based upon the benefits and risks to the patient, as well as financial considerations of the owner,
- formulate and effectively deliver a communication plan that includes the potential benefits and risks of the surgical procedures, as well as potential complications and costs to the owner,
- identify appropriate resource(s) to consult before performing an unfamiliar surgical procedure,
- recognize cases that require surgical expertise beyond the level possessed by the graduate and therefore require consultation and/or referral.

cc BY-NC This work is licensed under a Creative Commons Attribution-NonCommercial 4.0 International

4.3 The graduate will apply the standards of high-quality postoperative patient care in all common domestic species.

***** This rubric defines competencies for each NGO that students should complete in each year of the curriculum*****

Competency Rubric for each Year of the Curriculum	1VM	2VM	3VM	4VM
	Level of competence following completion of year 1.	*Level of competence following completion of year 2.*	*Level of competence following completion of year 3.*	*Expected level of competence at graduation.*
**4.3**<br>Apply the standards of pre- and post-operative patient care in all common domestic species.		**2A.** recognize the needs of the pre- and postoperative patient<br><br>**2B.** demonstrate pre- and postoperative care techniques associated with entry-level surgical interventions in common domestic species	**3A.** perform pre- and postoperative patient care in all common domestic species in a clinical scenario	**4A.** perform pre- and postoperative patient care in clinical patients of all common domestic species

Examples of session-level/meeting-level/unit-level outcomes to use as a guide to fulfill **NGO 4.3**:

- recognize the importance of postoperative support and pain management to achieving successful surgical outcomes,
- develop a postoperative patient plan that includes postoperative monitoring and the need for additional diagnostics or therapies,
- use knowledge of anatomy and physiology to monitor patients postoperatively,
- recognize the ongoing needs of the postoperative patient and provide appropriate postoperative and wound management to facilitate patient recovery,
- communicate with all members of the health care team to ensure a standard level of patient care within the practice,
- determine the appropriate length of hospitalization based upon the surgical procedure, as well as the patient status,
- recognize the need for an advanced home care plan that may include physical rehabilitation and pain management,
- formulate and communicate an effective homecare plan based on the ongoing needs of the patient and the ability of the owner to comply with recommendations,
- formulate an appropriate recheck plan for each common surgical procedure,
- communicate effectively with specialists who have performed surgery to coordinate appropriate postoperative patient care.

(cc) BY-NC This work is licensed under a Creative Commons Attribution-NonCommercial 4.0 International

NGO 5: Basic medicine skills, experience and case management

5.1 The graduate will safely, consistently, and effectively perform the basic medical procedures required to successfully manage common medical conditions in common domestic species.

***** This rubric defines competencies for each NGO that students should complete in each year of the curriculum*****

Competency Rubric for each Year of the Curriculum	1VM	2VM	3VM	4VM
	Level of competence following completion of year 1.	*Level of competence following completion of year 2.*	*Level of competence following completion of year 3.*	*Expected level of competence at graduation.*
**5.1**<br>Perform the medical procedures required to successfully manage common medical conditions in common domestic species.	**1A.** explain physiological conditions relative to common medical procedures<br><br>**1B.** locate and describe the regional anatomy relevant to common medical procedures<br><br>**1C.** perform selected medical procedures for common domestic species	**2A.** perform selected medical procedures for common domestic species	**3A.** perform the medical procedures required to manage common medical conditions in clinical scenarios	**4A.** perform the medical procedures required to manage common medical conditions in clinical patients

Examples of session-level/meeting-level/unit-level outcomes to use as a guide to fulfill **NGO 5.1**:

- locate and describe the regional anatomy relevant to common medical procedures,
- select appropriate medical procedures, taking into consideration the benefits and risks to the patient, owner finances, as well as the safety of the patient, owner, and other members of the health care team,
- describe and perform the basic medical procedures on patients safely and effectively.

(cc) BY-NC This work is licensed under a Creative Commons Attribution-NonCommercial 4.0 International

5.2 The graduate will formulate and implement appropriate medical treatment plans based upon patient findings to address the acute and long-term needs in all common domestic species.

***** This rubric defines competencies for each NGO that students should complete in each year of the curriculum*****

Competency Rubric for each Year of the Curriculum	1VM	2VM	3VM	4VM
	Level of competence following completion of year 1.	*Level of competence following completion of year 2.*	*Level of competence following completion of year 3.*	*Expected level of competence at graduation.*
**5.2**<br>Formulate and implement medical treatment and prevention plans to address the acute and long-term needs in all common domestic species.	**1A.** explain physiological processes that may be altered by medical treatment	**2A.** recognize and explain abnormal patient findings in common domestic species<br><br>**2B.** explain abnormal physiological processes that may require medical treatment<br><br>**2C.** identify treatment options based on patient findings	**3A.** formulate and implement medical treatment and prevention plans to address the acute and long-term needs in a clinical scenario<br><br>**3B.** communicate medical treatment and prevention plans options to clients in clinical scenarios	**4A.** formulate and implement medical treatment and prevention plans to address the acute and long-term needs in a clinical patient<br><br>**4B.** communicate medical treatment and prevention plans options to clients

Examples of session-level/meeting-level/unit-level outcomes to use as a guide to fulfill **NGO 5.2**:

- list and describe the common and significant diseases that are developmental, degenerative, metabolic, nutritional, neoplastic, infectious, immune mediated, toxic, and traumatic in nature for each of the common domestic species,
- explain the pathophysiology of common and significant diseases and recognize and explain the associated gross and histopathology lesions that may occur in the major organ systems,
- describe the normal and abnormal functions of the immune system and apply knowledge of immunology to formulate medical treatment plans,
- assess the grade and/or stage of disease in order to predict disease course and prognosis,
- formulate a comprehensive medical treatment plan to address the acute and long-term needs of the patient,
- utilize common veterinary products in an appropriate manner in order to implement a comprehensive medical treatment plan,
- formulate an appropriate dietary plan to meet the acute and long-term needs of the patient,
- use knowledge of physiology to monitor patient response to medical treatment,
- predict potential complications of the medical treatment plan and respond appropriately to complications when they arise,
- formulate and effectively communicate a homecare and recheck plan based on the ongoing needs of the patient and the ability of the owner to comply with recommendations.

(cc) BY-NC This work is licensed under a Creative Commons Attribution-NonCommercial 4.0 International

5.3 The graduate will formulate and implement an appropriate behavioral treatment plan based upon patient history and clinical findings to address the acute and long-term behavioral needs in common domestic species.

***** This rubric defines competencies for each NGO that students should complete in each year of the curriculum*****

Competency Rubric for each Year of the Curriculum	1VM	2VM	3VM	4VM
	Level of competence following completion of year 1.	*Level of competence following completion of year 2.*	*Level of competence following completion of year 3.*	*Expected level of competence at graduation.*
**5.3**<br>Formulate and implement a behavioral treatment plan based upon patient history and clinical findings to address the acute and long-term behavioral needs in common domestic species.	**1A.** recognize normal behaviors in common domestic species<br><br>**1B.** list abnormal behaviors in common domestic species	**2A.** recognize abnormal behaviors in common domestic species<br><br>**2B.** distinguish the origin of abnormal behaviors in common domestic species<br><br>**2C.** identify behavioral concerns in common domestic species in a clinical scenario	**3A.** formulate a behavioral treatment plan based upon patient history and clinical findings to address the acute and long-term behavioral needs in a clinical scenario	**4A.** formulate a behavioral treatment plan based upon patient history and clinical findings to address the acute and long-term behavioral needs in common domestic species in a clinical patient

Examples of session-level/meeting-level/unit-level outcomes to use as a guide to fulfill **NGO 5.3**:

- describe the anatomical variations in the sense organs of common domestic species and their impact on sensory perception,
- describe the principles of animal learning and explain how learning relates to normal and abnormal animal behavior,
- recognize normal animal behaviors within common management practices,
- diagnose common behavioral concerns identified by owners,
- describe medical and behavioral components that mimic one another,
- formulate a comprehensive treatment plan to address common behavioral problems,
- communicate a treatment plan to the owner that addresses the ongoing needs of the patient and the ability of the owner to comply with recommendations.

cc BY-NC This work is licensed under a Creative Commons Attribution-NonCommercial 4.0 International

NGO 6: Emergency and intensive care case management

6.1 The graduate will recognize the clinical signs associated with a patient emergency in all common domestic species and formulate an appropriate emergency protocol in order to rapidly assess and stabilize the patient.

***** This rubric defines competencies for each NGO that students should complete in each year of the curriculum*****

Competency Rubric for each Year of the Curriculum	1VM	2VM	3VM	4VM
	Level of competence following completion of year 1.	*Level of competence following completion of year 2.*	*Level of competence following completion of year 3.*	*Expected level of competence at graduation.*
**6.1**<br>Recognize the clinical signs associated with a patient emergency in common domestic species and formulate an emergency protocol to assess and stabilize the patient.	**1A.** prioritize components of the physical examination in the emergent patient<br><br>**1B.** recognize clinical signs associated with the emergent patient<br><br>**1C.** define common medical terminology associated with the emergent patient	**2A.** identify interventions associated with the emergent patient<br><br>**2B.** categorize emergent status	**3A.** recognize the clinical signs associated with a patient emergency in common domestic species<br><br>**3B.** formulate an emergency protocol to assess and stabilize the patient in a clinical scenario	**4A.** recognize the clinical signs associated with a patient emergency in common domestic species<br><br>**4B.** formulate an emergency protocol to assess and stabilize the patient in a clinical case

Examples of session-level/meeting-level/unit-level outcomes to use as a guide to fulfill **NGO 6.1**:

- use knowledge of anatomy and physiology to recognize a patient emergency based upon physical examination and clinical findings,
- categorize the type of medical emergency according to the type of problem and plan accordingly,
- recall the common medical procedures used to stabilize patients in critical condition and clearly explain the indications and limitations of each procedure,
- classify common therapeutic veterinary products that can be used to stabilize patients and explain their mechanisms of action, pharmacologic properties, and physiologic effects on the patient,
- formulate an appropriate treatment plan to attempt patient stabilization, taking into account the benefits and risks, prognosis, and financial considerations of the owner,
- recognize when patient stabilization is not possible and effectively communicate the option of euthanasia with the owner,
- design a triage protocol for the health care team that provides for rapid assessment and prioritization of the medical needs of multiple patients.

BY-NC This work is licensed under a Creative Commons Attribution-NonCommercial 4.0 International

6.2 The graduate will apply an emergency treatment plan to stabilize a patient in critical condition, including safely, consistently, and effectively performing basic emergency procedures.

***** This rubric defines competencies for each NGO that students should complete in each year of the curriculum*****

Competency Rubric for each Year of the Curriculum	1VM	2VM	3VM	4VM
	Level of competence following completion of year 1.	*Level of competence following completion of year 2.*	*Level of competence following completion of year 3.*	*Expected level of competence at graduation.*
**6.2**<br>Apply a treatment plan to stabilize a patient in critical condition, and perform emergency procedures.	**1A.** prioritize affected physiologic systems in the emergent patient<br><br>**1B.** identify and describe anatomical structures associated with common emergency procedures	**2A.** perform components of emergency procedures in a clinical scenario<br><br>**2B.** develop a stabilization plan for an emergent patient<br><br>**2C.** communicate with others during an emergency clinical scenario<br><br>**2D.** describe the management of the healthcare team in an emergency situation	**3A.** apply a treatment plan to stabilize a patient in critical condition, and perform emergency procedures in a clinical scenario<br><br>**3B.** manage a healthcare team in an emergency clinical scenario<br><br>**3C.** communicate with others during an emergency clinical scenario	**4A.** apply a treatment plan to stabilize a patient in critical condition, and perform emergency procedures in a clinical patient<br><br>**4B.** communicate with others during an emergency situation

Examples of session-level/meeting-level/unit-level outcomes to use as a guide to fulfill **NGO 6.2**:

- locate and describe the regional anatomy relevant to performing basic emergency procedures,
- demonstrate the proper technique to safely, consistently, and effectively perform all basic emergency procedures used to stabilize patients,
- implement a comprehensive treatment plan in order to successfully manage a patient in critical condition,
- manage a health care team effectively during a patient emergency,
- communicate with owners effectively to present treatment options for a patient in critical condition, including discussing the potential benefits and risks of the procedure(s), prognosis, and costs,
- communicate with owners effectively on a regular basis in order to keep them well informed during a dynamic emergency situation.

BY-NC This work is licensed under a Creative Commons Attribution-NonCommercial 4.0 International

6.3 The graduate will recognize patient conditions that require intensive care, appraise the level of care required, and implement appropriate treatment protocols, including managing the transfer or referral of a patient to another facility when indicated.

***** This rubric defines competencies for each NGO that students should complete in each year of the curriculum*****

Competency Rubric for each Year of the Curriculum	1VM	2VM	3VM	4VM
	Level of competence following completion of year 1.	*Level of competence following completion of year 2.*	*Level of competence following completion of year 3.*	*Expected level of competence at graduation.*
**6.3**<br>Recognize patient conditions that require critical care, appraise the level of care required, and implement treatment protocols, including managing the transfer or referral of a patient to another facility when indicated.	**1A.** demonstrate technical skills associated with treatment protocols<br><br>**1B.** identify components of a written patient referral document	**2A.** describe resources necessary to provide critical care<br><br>**2B.** synthesize physical examination and diagnostic results to provide ongoing patient management in a clinical scenario<br><br>**2C.** recognize the pathophysiologic processes associated with patients in critical care<br><br>**2D.** complete a written patient referral document	**3A.** recognize patient conditions that require critical care, appraise the level of care required, design treatment protocols, including managing the transfer or referral of a patient to another facility when indicated in a clinical scenario<br><br>**3B.** determine when referral is necessary based on available resources for a patient requiring critical care in a clinical scenario<br><br>**3C.** communicate patient transfer with referral hospital, client and veterinarian through both oral and written communication in a clinical scenario	**4A.** recognize patient conditions that require critical care, appraise the level of care required, and implement treatment protocols, including managing the transfer or referral of a patient to another facility when indicated in a clinical patient

Examples of session-level/meeting-level/unit-level outcomes to use as a guide to fulfill **NGO 6.3**:

- use knowledge of anatomy and physiology to differentiate between patients that require intensive care and basic care during hospitalization, based upon the physical examination and other clinical findings,
- list the parameters that are used to monitor patients at regular intervals during hospitalization in intensive care,
- classify common therapeutic veterinary products used to manage patients requiring intensive care and explain their mechanisms of action, pharmacologic properties, and physiologic effects on the patient,
- formulate an appropriate treatment plan for a patient that requires intensive care taking into consideration the potential risks and benefits, prognosis, and financial considerations of the owner,
- communicate with the owner to present treatment options for a patient requiring intensive care, including discussing the severity of disease, prognosis, potential benefits and risks of any procedure(s), and the costs,
- implement a treatment plan for a patient requiring intensive care during hospitalization, including managing a health care team in order to fully meet the needs of the patient,
- utilize professional guidelines and assess personal professional competency level to judge when it is appropriate to refer a patient.

cc BY-NC This work is licensed under a Creative Commons Attribution-NonCommercial 4.0 International

NGO 7: Health promotion, disease prevention/biosecurity, zoonosis, and food safety

7.1 The graduate will evaluate risk factors for individual or groups of animals for medical conditions in common domestic species.

***** This rubric defines competencies for each NGO that students should complete in each year of the curriculum*****

Competency Rubric for each Year of the Curriculum	1VM	2VM	3VM	4VM
	Level of competence following completion of year 1.	*Level of competence following completion of year 2.*	*Level of competence following completion of year 3.*	*Expected level of competence at graduation.*
**7.1** Evaluate risk factors for individual or groups of animals for medical conditions in common domestic species.	**1A.** define risk factors for disease in common domestic species	**2A.** identify the presence of risk factors for disease<br><br>**2B.** explain methods to mitigate risk factors of disease	**3A.** evaluate risk factors for individual or groups of animals for medical conditions in common domestic species in a clinical scenario	**4A.** evaluate risk factors for individual or groups of animals for medical conditions in common domestic species in a clinical patient

Examples of session-level/meeting-level/unit-level outcomes to use as a guide to fulfill **NGO 7.1**:

- list the risk factors for common and significant diseases that are developmental, degenerative, metabolic, nutritional, neoplastic, infectious, immune mediated, toxic, and traumatic in nature,
- interpret the signalment (i.e., age, sex, breed, and function), patient history, and other conditions to identify the presence of risk factors for disease,
- explain the methods for mitigating or eliminating risk factors for disease.

(cc) BY-NC This work is licensed under a Creative Commons Attribution-NonCommercial 4.0 International

7.2 The graduate will design and implement preventive programs, encompassing biosecurity and animal husbandry, to improve the health and well-being of individual animals and animal populations.

***** This rubric defines competencies for each NGO that students should complete in each year of the curriculum*****

Competency Rubric for each Year of the Curriculum	1VM	2VM	3VM	4VM
	Level of competence following completion of year 1.	*Level of competence following completion of year 2.*	*Level of competence following completion of year 3.*	*Expected level of competence at graduation.*
**7.2** Design and implement preventive programs, encompassing biosecurity and animal husbandry, to improve the health and well-being of individual animals and animal populations.	**1A.** recognize and classify common bacterial, viral and fungal pathogens of animals<br><br>**1B.** describe the function of the immune system and its role in designing effective disease prevention programs<br><br>**1C.** recognize the role of the environment in animal husbandry<br><br>**1D.** explain the unique nutritional needs for common domestic species	**2A.** describe common pathogens of animals and their potential impact on animal health<br><br>**2B.** recognize and assess biosecurity risks in a defined environment, formulate a biosecurity and communication plan for a defined situation in a clinical scenario<br><br>**2C.** formulate nutritional plans that promote the health and well-being of common domestic species	**3A.** design preventive programs to improve the health and well-being of individual animals and animal populations in a clinical scenario<br><br>**3B.** develop and communicate a comprehensive preventive program to clients and the healthcare team in clinical scenarios	**4A.** implement preventive programs, encompassing biosecurity and animal husbandry, to improve the health and well-being of clinical patients<br><br>**4B.** develop and communicate a comprehensive preventive program to clients and the healthcare team for clinical patients

Examples of session-level/meeting-level/unit-level outcomes to use as a guide to fulfill **NGO 7.2**:

- recognize and classify important viral, bacterial, fungal, and parasitic pathogens of animals,
- relate pathogens with common infectious diseases and understand their potential impact on population health,
- describe the function of the immune system and apply their knowledge of immunology to design disease prevention programs addressing animal health and husbandry,
- classify common therapeutic veterinary products used in preventive programs and explain their mechanisms of action, pharmacologic properties, and physiologic effects on animals,
- design vaccination and parasite management and prevention programs for individual animals and animal populations,
- explain the unique nutritional needs of each of the common domestic species and formulate nutritional plans that promote the health and well-being of animals,1
- recognize the role of epidemiologic study design and its application to disease prevention and control,
- explain the role of veterinarians in safeguarding against agroterrorism and bioterrorism,
- identify diseases of regulatory importance,
- recognize the principles of biosecurity, including sanitary methods, the movement of animals and people, immunity, and biocontainment,
- assess the biosecurity risks in a defined environment, formulate a biosecurity plan for a defined situation, and communicate the appropriate biosecurity protocol to clients or the public,
- formulate a comprehensive preventive plan, taking into consideration feasibility of implementation, owner finances, and owner compliance,
- communicate to owners (in writing and orally) a comprehensive preventive plan in a manner that is readily understood and promotes adherence to recommendations,
- work with appropriate regulatory agencies to participate in an epidemiological inquiry in case of occurrence of a reportable disease, including collection, handling, and transport of appropriate specimens or samples.

(cc) BY-NC This work is licensed under a Creative Commons Attribution-NonCommercial 4.0 International

7.3 The graduate will recognize the potential for zoonoses and communicate evidence-based recommendations to clients and the healthcare team to minimize the risk of zoonotic disease transmission.

***** This rubric defines competencies for each NGO that students should complete in each year of the curriculum*****

Competency Rubric for each Year of the Curriculum	1VM	2VM	3VM	4VM
	Level of competence following completion of year 1.	*Level of competence following completion of year 2.*	*Level of competence following completion of year 3.*	*Expected level of competence at graduation.*
**7.3**<br>Recognize the potential for zoonosis and communicate evidence-based recommendations to clients and the healthcare team to minimize the risk of zoonotic disease transmission.	**1A.** list zoonotic pathogens<br><br>**1B.** identify sources of information to support current evidence-based recommendations for zoonotic diseases	**2A.** explain pathogenesis associated with common zoonotic diseases<br><br>**2B.** recognize human-specific risks for zoonosis	**3A.** recognize the potential for zoonosis in a clinical patient/group of animals<br><br>**3B.** Recognize and communicate a zoonotic disease prevention plan to clients and the healthcare team in a clinical scenario	**4A.** recognize the potential for zoonosis in a clinical patient<br><br>**4B.** communicate evidence-based recommendations to clients and the healthcare team to minimize the risk of zoonotic disease transmission

Examples of session-level/meeting-level/unit-level outcomes to use as a guide to fulfill **NGO 7.3**:

- list the important zoonotic pathogens,
- identify the clinical signs, clinical course, transmission potential, and pathogen associated with common zoonotic diseases,
- recognize client-specific risks for zoonoses,
- formulate a customized zoonosis prevention plan for their clients,
- communicate and document in the medical record a zoonotic disease prevention plan in a manner that is readily understood and promotes client adherence to recommendations.

(cc) BY-NC This work is licensed under a Creative Commons Attribution-NonCommercial 4.0 International

7.4 The graduate will apply knowledge of common foodborne pathogens to effectively promote and maintain a safe food supply.

***** This rubric defines competencies for each NGO that students should complete in each year of the curriculum*****

Competency Rubric for each Year of the Curriculum	1VM	2VM	3VM	4VM
	Level of competence following completion of year 1.	*Level of competence following completion of year 2.*	*Level of competence following completion of year 3.*	*Expected level of competence at graduation.*
**7.4**<br>Apply knowledge of common foodborne pathogens to promote and maintain a safe food supply.	**1A.** list common foodborne pathogens	**2A.** explain recommended food safety practices for production facilities in a clinical scenario<br><br>**2B.** communicate with stakeholders about risk management, control and prevention of foodborne pathogens in a clinical scenario	**3A.** apply knowledge of common foodborne pathogens to promote and maintain a safe food supply	**4A.** explain recommended food safety practices for production facilities<br><br>**4B.** communicate with stakeholders/producers about risk management, control and prevention of foodborne pathogens for clinical cases

Examples of session-level/meeting-level/unit-level outcomes to use as a guide to fulfill **NGO 7.4**:

- explain on-farm food safety practices,
- demonstrate knowledge of state and federal regulations regarding food safety and inspection,
- list the important foodborne pathogens and link the pathogens to important food sources,
- recommend appropriate diagnostic testing measures to identify the source of the pathogen in a foodborne outbreak,
- analyze a client's risk and formulate a customized prevention plan for foodborne disease,
- explain the purpose and function of a HACCP plan and assess the efficacy of a HACCP plan,
- communicate effectively with clients, producers, and regulatory personnel about risk management, control, and prevention of foodborne pathogens.

This work is licensed under a Creative Commons Attribution-NonCommercial 4.0 International

7.5 The graduate will apply knowledge of noninfectious adulterants in the human and animal food supply to effectively promote and maintain a safe food supply.

***** This rubric defines competencies for each NGO that students should complete in each year of the curriculum*****

Competency Rubric for each Year of the Curriculum	1VM	2VM	3VM	4VM
	Level of competence following completion of year 1.	*Level of competence following completion of year 2.*	*Level of competence following completion of year 3.*	*Expected level of competence at graduation.*
**7.5** The graduate will apply knowledge of noninfectious adulterants in the human and animal food supply to effectively promote and maintain a safe food supply.	**1A.** define physical and chemical adulterants as it pertains to human and animal food supply	**2A.** formulate drug treatment plans in clinical scenarios that support the judicious use of pharmaceuticals (drug residue, withdrawal times, antimicrobial resistance)<br><br>**2B.** apply knowledge of chemical and physical adulterants in a clinical scenario	**3A.** communicate with stakeholders to promote compliance with food safety standards in a clinical scenario	**4A.** formulate drug treatment plans for clinical patients that support the judicious use of pharmaceuticals (drug residue, withdrawal times, antimicrobial resistance)

Examples of session-level/meeting-level/unit-level outcomes to use as a guide to fulfill **NGO 7.5**:

- explain the principles of pharmacokinetics that are applied to develop drug withdrawal times,
- recognize the potential risks of animal feed contamination with biological/chemical adulterants,
- formulate drug treatment plans that support the judicious use of antimicrobials to minimize antibiotic resistance,
- communicate effectively with clients and producers in order to promote compliance with food safety standards.

(cc) BY-NC This work is licensed under a Creative Commons Attribution-NonCommercial 4.0 International

7.6 The graduate will participate in transdisciplinary efforts to safeguard human, environmental, and animal health to demonstrate the veterinary role in the One Health triad.

***** This rubric defines competencies for each NGO that students should complete in each year of the curriculum*****

Competency Rubric for each Year of the Curriculum	1VM	2VM	3VM	4VM
	Level of competence following completion of year 1.	*Level of competence following completion of year 2.*	*Level of competence following completion of year 3.*	*Expected level of competence at graduation.*
**7.6** Participate in interdisciplinary efforts to safeguard human, environmental, and animal health to demonstrate the veterinary role in the One Health triad.	**1A.** define the importance of One Health medicine	**2A.** describe the role veterinarians play in the One Health triad	**3A.** identify interdisciplinary efforts to safeguard human, environmental, and animal health	**4A.** participate in interdisciplinary efforts to safeguard human, environmental, and animal health to demonstrate the veterinary role in the One Health triad

Examples of session-level/meeting-level/unit-level outcomes to use as a guide to fulfill **NGO 7.6**:

- discuss the global economic, cultural, and environmental impacts and animal and human health-related issues associated with animal agriculture,
- explain the role of veterinarians in interacting with physicians, public health practitioners, and risk analysts to ensure safe food,
- explain the role of the veterinarian in disaster management,
- work within the federal, state-, and local-level framework for the development and implementation of disaster management plans,
- develop a personal disaster preparedness plan for their home or clinical practice,
- recognize the role of the veterinarian as an animal expert in the community,
- explain common mechanisms leading to the development of antimicrobial resistance in common pathogens,
- provide leadership to society on ethical considerations involved in the use and care of animals by humans.

BY-NC This work is licensed under a Creative Commons Attribution-NonCommercial 4.0 International

NGO 8: Client communications and ethical conduct

8.1 The graduate will communicate with clients, colleagues, staff, and the general public through both oral and written communication, using appropriate professional language.

***** This rubric defines competencies for each NGO that students should complete in each year of the curriculum*****

Competency Rubric for each Year of the Curriculum	1VM	2VM	3VM	4VM
	Level of competence following completion of year 1.	*Level of competence following completion of year 2.*	*Level of competence following completion of year 3.*	*Expected level of competence at graduation.*
**8.1**<br>Communicate with clients, colleagues, staff, and the general public through both oral and written communication, using appropriate professional language.	**1A.** recognize and demonstrate core communication skills in low risk team and client-based scenarios<br><br>**1B.** communicate in oral and written form using appropriate communication skills, and scientific and professional language	**2A.** communicate in oral and written form in challenging clinical scenarios with clients, colleagues, staff, and general public using appropriate communication skills, and scientific and professional language	**3A.** communicate in oral and written form in high risk clinical scenarios with clients, colleagues, staff, and general public using appropriate communication skills, and scientific and professional language	**4A.** communicate with clients, colleagues, staff, and the general public through both oral and written communication, using appropriate scientific and professional language

Examples of session-level/meeting-level/unit-level outcomes to use as a guide to fulfill **NGO 8.1**:

- explain the role of client education in providing high-quality patient care,
- describe the precepts of good verbal and nonverbal communication techniques,
- apply the concept of veterinarian-client-patient relationship in order to maintain appropriate client confidentiality,
- list several channels veterinarians can utilize to communicate with their clients,
- adjust their language to the level of education or experience of the client when communicating with a client,
- demonstrate situational awareness to modulate their verbal and nonverbal communication as needed in order to be congruent with the situation,
- develop and implement an effective client communication plan, including providing instructions for long-term patient care,
- develop and implement a multichannel public relations plan for the general public.

This work is licensed under a Creative Commons Attribution-NonCommercial 4.0 International

8.2 The graduate will demonstrate professional conduct.

***** This rubric defines competencies for each NGO that students should complete in each year of the curriculum*****

Competency Rubric for each Year of the Curriculum	1VM	2VM	3VM	4VM
	Level of competence following completion of year 1.	*Level of competence following completion of year 2.*	*Level of competence following completion of year 3.*	*Expected level of competence at graduation.*
**8.2** Demonstrate professional conduct.	**1A.** demonstrate honesty, integrity, and fairness in interactions with others<br><br>**1B.** describe self-awareness and self-management<br><br>**1C.** analyze situations that pose ethical dilemmas, identify resources and develop an appropriate response	**2A.** demonstrate professional conduct<br><br>**2B.** recognize inappropriate behavior and suggest possible corrective actions	**3A.** demonstrate professional conduct<br><br>**3B.** recognize and take corrective action for inappropriate behavior	**4A.** demonstrate professional conduct with clients, staff, colleagues, and general public<br><br>**4B.** recognize and take corrective action for inappropriate behavior

Examples of session-level/meeting-level/unit-level outcomes to use as a guide to fulfill **NGO 8.2**:

- demonstrate honesty, integrity, and fairness in interactions with colleagues and clients,
- recognize the importance of conducting themselves in an ethical manner in order to maintain the overall well-being of the veterinary profession,
- recognize situations that pose a potential conflict of interest and take appropriate measures to avoid or resolve the conflict,
- demonstrate professional behavior in a consistent manner and recognize inappropriate behavior in others that requires corrective action,
- identify situations that may pose an ethical dilemma and take measures to avoid or resolve the issue,
- recognize a professional error and take appropriate corrective measures to reduce or prevent future errors,
- communicate a professional mistake to their client and/or health care team and choose appropriate corrective actions,
- recognize the ethical responsibility to provide essential services to save lives and relieve animal suffering.

This work is licensed under a Creative Commons Attribution-NonCommercial 4.0 International

NGO 9: Critical analysis of new information and research findings relevant to veterinary medicine

9.1 The graduate will identify, review, and critically evaluate biomedical literature and apply it to the practice of contemporary, evidence-based veterinary medicine.

***** This rubric defines competencies for each NGO that students should complete in each year of the curriculum*****

Competency Rubric for each Year of the Curriculum	1VM	2VM	3VM	4VM
	Level of competence following completion of year 1.	*Level of competence following completion of year 2.*	*Level of competence following completion of year 3.*	*Expected level of competence at graduation.*
**9.1**<br>Identify, review, and critically evaluate biomedical literature and apply it to the practice of contemporary, evidence-based veterinary medicine.	**1A.** recognize the principles of evidence-based medicine and describe their importance<br><br>**1B.** identify a personal knowledge gap and utilize appropriate resources to address it	**2A.** recognize the principles of evidence-based medicine and evaluate a clinical scenario based on them<br><br>**2B.** formulate a relevant question to address a personal knowledge gap and utilize appropriate resources to address it<br><br>**2C.** critically appraise the relevant information and published evidence by applying knowledge of study design	**3A.** utilize the principles of evidence-based medicine in a clinical scenario	**4A.** identify, review, and critically evaluate biomedical literature and apply it to the practice of contemporary, evidence-based veterinary medicine

Examples of session-level/meeting-level/unit-level outcomes to use as a guide to fulfill **NGO 9.1**:

- recognize the importance of evidence-based medicine and apply its principles to the practice of veterinary medicine,
- identify a knowledge gap, formulate a relevant question to address the knowledge gap and identify and utilize appropriate resources (e.g., PubMed, CAB Abstracts) to address the knowledge gap,
- critically appraise the relevant information and published evidence,
- integrate and apply new knowledge to the practice of veterinary medicine.

CC BY-NC This work is licensed under a Creative Commons Attribution-NonCommercial 4.0 International

9.2 The graduate will demonstrate the skills necessary to maintain life-long learning and the need to pursue professional development in order to maintain competency throughout the duration of their career.

***** This rubric defines competencies for each NGO that students should complete in each year of the curriculum*****

Competency Rubric for each Year of the Curriculum	1VM	2VM	3VM	4VM
	Level of competence following completion of year 1.	*Level of competence following completion of year 2.*	*Level of competence following completion of year 3.*	*Expected level of competence at graduation.*
**9.2**<br>Demonstrate the skills necessary to maintain life-long learning and explain the value of pursuing professional development in order to maintain competency throughout the duration of a veterinary career.	**1A.** discuss the variety of career opportunities (in addition to clinical practice) and the requirements for pursuit of each	**2A.** identify available resources to maintain future professional competency	**3A.** reflect on desired career path and the requirements for pursuit of each<br><br>**3B.** apply previous learning to novel clinical scenarios<br><br>**3C.** apply new knowledge to common clinical scenarios	**4A.** explain the requirements to obtain and maintain veterinary licensure and accreditation<br><br>**4B.** describe opportunities to maintain future professional competency<br><br>**4C.** transfer previous learning to novel clinical situations

Examples of session-level/meeting-level/unit-level outcomes to use as a guide to fulfill **NGO 9.2**:

- recognize the role of basic and applied research to the advancement of veterinary medicine,
- utilize the available resources (e.g., TAMU Medical Sciences Library, TAMU Office of Continuing Education) to be able to maintain future professional competency,
- recognize the variety of career opportunities, in addition to clinical practice (e.g., public health, industry, government employment),
- recognize opportunities to pursue advanced training in specialty areas,
- recognize the need to obtain and maintain veterinary licensure and perform the requirements (including continuing education) to obtain and maintain licensure,
- recognize the professional obligations of state and federal accreditation and perform the requirements to obtain and maintain accreditation.

cc BY-NC This work is licensed under a Creative Commons Attribution-NonCommercial 4.0 International

NGO 10: Team collaboration, leadership, and practice management

10.1 The graduate will operate effectively with other members of a health care team and the general public.

***** This rubric defines competencies for each NGO that students should complete in each year of the curriculum*****

Competency Rubric for each Year of the Curriculum	1VM	2VM	3VM	4VM
	Level of competence following completion of year 1.	*Level of competence following completion of year 2.*	*Level of competence following completion of year 3.*	*Expected level of competence at graduation.*
**10.1** Collaborate effectively with other members of a health care team and the general public.	**1A.** collaborate with peers and mentors<br><br>**1B.** provide balanced, descriptive feedback to peers	**2A.** collaborate with health care team<br><br>**2B.** utilize feedback to enhance team performance	**3A.** collaborate with health care teams in a professional setting	**4A.** collaborate with other members of a health care team and the general public

Examples of session-level/meeting-level/unit-level outcomes to use as a guide to fulfill **NGO 10.1**:

- demonstrate professional behavior consistently when interacting with other members of the health care team,
- communicate with a broad range of people, adjusting their language according to the level of education or experience of the audience,
- evaluate other members of the health care team and provide appropriate feedback to ensure professional behavior with clients and colleagues,
- demonstrate confidentiality when handling knowledge and information about others in the professional setting.

BY-NC This work is licensed under a Creative Commons Attribution-NonCommercial 4.0 International

10.2 The graduate will organize their own professional activities and manage other members of the healthcare team in order to provide optimal patient care.

***** This rubric defines competencies for each NGO that students should complete in each year of the curriculum*****

Competency Rubric for each Year of the Curriculum	1VM	2VM	3VM	4VM
	Level of competence following completion of year 1.	*Level of competence following completion of year 2.*	*Level of competence following completion of year 3.*	*Expected level of competence at graduation.*
**10.2**<br>Organize professional activities and manage other members of the healthcare team in order to provide optimal patient care.	**1A.** demonstrate organizational skills in an academic setting to enhance personal performance<br><br>**1B.** explain the leadership opportunities and responsibilities of veterinarians in society<br><br>**1C.** identify leadership qualities that enhance team performance	**2A.** develop a personal plan to strengthen leadership and management skills	**3A.** demonstrate organizational skills in a professional setting<br><br>**3B.** lead members of the healthcare team in clinical scenarios	**4A.** lead members of the healthcare team to provide optimal patient care in the professional setting

Examples of session-level/meeting-level/unit-level outcomes to use as a guide to fulfill **NGO 10.2**:

- explain the value of leadership to the veterinary profession and describe some attributes common to effective leaders,
- demonstrate leadership skills, including organizing oneself and others, in order to appropriately manage a patient,
- appraise their leadership strengths and weaknesses and identify experiences to broaden their own professional development,
- explain the unique leadership opportunities and responsibilities of veterinarians with regard to patients, clients, and society,
- explain the importance of organized veterinary medicine to the profession.

This work is licensed under a Creative Commons Attribution-NonCommercial 4.0 International

10.3 The graduate will apply the principles of practice management to the running of a veterinary practice as a business.

***** This rubric defines competencies for each NGO that students should complete in each year of the curriculum*****

Competency Rubric for each Year of the Curriculum	1VM	2VM	3VM	4VM
	Level of competence following completion of year 1.	*Level of competence following completion of year 2.*	*Level of competence following completion of year 3.*	*Expected level of competence at graduation.*
**10.3** Apply the principles of veterinary practice management.	**1A.** explain the fundamental elements of practice management and its importance to high quality patient care	**2A.** identify strategies to apply the fundamental elements of practice management in a professional setting	**3A.** develop a practice management plan within clinical scenarios	**4A.** demonstrate the principles of veterinary practice management

Examples of session-level/meeting-level/unit-level outcomes to use as a guide to fulfill **NGO 10.3**:

- recognize the importance of managing personal finances and the impact that lifestyle choices have on student debt load,
- prepare a personal budget through which to manage their finances,
- explain the basic veterinary business performance functions,
- explain the pros and cons of different business designs, including sole proprietorship, partnerships, corporation, and limited liability company,
- identify the major components of business overhead,
- explain the role of personal and business insurance (e.g., health insurance, disability insurance, workers' compensation, and malpractice insurance) in safeguarding financial assets,
- explain the fundamental elements required to provide high-quality customer service to clients,
- describe the selection criteria for the ideal veterinary associate,
- identify their professional priorities and needs and use this information to negotiate a career opportunity and/or adjust their budget,
- analyze a business plan to determine the break-even point for new equipment or other financial investments within their practice.

(cc) BY-NC This work is licensed under a Creative Commons Attribution-NonCommercial 4.0 International

NGO 11: Multicultural awareness and personal wellness

11.1 The graduate will demonstrate sensitivity to people who are different from them and interact with others free of discrimination in professional settings.

***** This rubric defines competencies for each NGO that students should complete in each year of the curriculum*****

Competency Rubric for each Year of the Curriculum	1VM	2VM	3VM	4VM
	Level of competence following completion of year 1.	*Level of competence following completion of year 2.*	*Level of competence following completion of year 3.*	*Expected level of competence at graduation.*
**11.1** Demonstrate sensitivity to diverse groups and interact free of discrimination with others in professional settings.	**1A.** describe common forms of bias or discrimination that might occur in a professional setting<br><br>**1B.** define diversity and list major categories in which people differ	**2A.** recognize bias or discrimination in professional settings<br><br>**2B.** explain the value of diversity in a professional setting	**3A.** describe potential corrective actions to mitigate bias or discrimination in a professional setting<br><br>**3B.** collaborate with a diverse population in a professional setting	**4A.** implement corrective actions to mitigate bias or discrimination in a professional setting<br><br>**4B.** collaborate with a diverse population in a clinical setting

Examples of session-level/meeting-level/unit-level outcomes to use as a guide to fulfill **NGO 11.1**:

- define the term diversity and list the major categories in which people differ from one another,
- explain the value of diversity and inclusiveness and its benefits to the veterinary profession,
- explain how the categories of diversity may influence thoughts and actions,
- describe their own story and explain how their own background and characteristics may influence their thoughts and actions,
- recognize the potential for bias and discrimination in the thoughts and actions in their professional life,
- implement corrective actions to reduce bias and discrimination towards people who are different from them,
- apply their understanding of diversity to professional decision-making,
- recognize bias and discrimination that exist in the professional organizations and entities in which they work.

This work is licensed under a Creative Commons Attribution-NonCommercial 4.0 International

11.2 The graduate will develop a capacity for effectively managing their own personal, physical, emotional, and financial needs in order to sustain high quality patient care and professional satisfaction throughout the duration of their career.

***** This rubric defines competencies for each NGO that students should complete in each year of the curriculum*****

Competency Rubric for each Year of the Curriculum	1VM	2VM	3VM	4VM
	Level of competence following completion of year 1.	*Level of competence following completion of year 2.*	*Level of competence following completion of year 3.*	*Expected level of competence at graduation.*
**11.2**<br>Develop a plan for managing physical, emotional, and financial needs to sustain professional competence and personal satisfaction for the duration of one's career.	**1A.** explain where to locate resources to manage wellness (e.g., counseling services, financial advisors)<br><br>**1B.** recognize when personal, physical, emotional, or financial needs are impaired, and identify steps to seek support<br><br>**1C.** describe strategies to maintain personal, physical, emotional, and financial wellness	**2A.** describe risk factors in the profession for various types of impairments including mental illness<br><br>**2B.** explain the impact of impairment on patient care and professional satisfaction<br><br>**2C.** recognize when personal, physical, emotional, or financial needs are impaired, and identify steps to seek support	**3A.** explain where to locate resources to manage wellness after graduation<br><br>**3B.** recognize when impaired physical, emotional, or financial needs are detrimentally affecting professional competence and personal satisfaction, and recognize corrective actions<br><br>**3C.** describe strategies to maintain personal, physical, emotional, and financial wellness	**4A.** develop a plan for managing physical, emotional, and financial needs to sustain professional competence and personal satisfaction for the duration of one's career

Examples of session-level/meeting-level/unit-level outcomes to use as a guide to fulfill **NGO 11.2**:

- describe the components of personal, physical, emotional and financial wellness,
- explain the role that personal, physical, emotional and financial wellness plays in maintaining high quality patient care and professional satisfaction,
- explain the unique professional circumstances of the veterinary profession that increase risk factors for impairment and/or mental illness (e.g., emotional burnout, compassion fatigue, access to controlled substances),
- develop a long-term plan to maintain personal, physical, emotional and financial wellness throughout their career,
- recognize when they have a personal, physical, emotional, or financial need,
- develop short- and long-term plans to address their needs, including recognition of sources of assistance,
- recognize when they or someone they work with is impaired or mentally ill and requires assistance and explain the appropriate steps in seeking the necessary support or help.

(cc) BY-NC This work is licensed under a Creative Commons Attribution-NonCommercial 4.0 International

NGO 12: Legal and regulatory compliance

12.1 The graduate will recognize the legal responsibilities of the veterinarian in relation to patients, clients, society, and the environment and operate within these professional parameters.

***** This rubric defines competencies for each NGO that students should complete in each year of the curriculum*****

Competency Rubric for each Year of the Curriculum	1VM	2VM	3VM	4VM
	Level of competence following completion of year 1.	*Level of competence following completion of year 2.*	*Level of competence following completion of year 3.*	*Expected level of competence at graduation.*
**12.1** Recognize the legal responsibilities of the veterinarian in relation to patients, clients, society, and the environment and operate within these professional parameters.	**1A.** describe the legal responsibilities of the veterinarian in relation to patients, clients, society, and the environment	**2A.** recognize the legal responsibilities of the veterinarian in relation to patients, clients, society, and the environment in a clinical scenario<br><br>**2B.** identify local and federal diseases of regulatory importance and describe how to report them to appropriate authorities	**3A.** describe compliance with legal regulations and recognize incidents of noncompliance<br><br>**3B.** identify local and federal diseases of regulatory importance and report them to appropriate authorities in a clinical scenario<br><br>**3C.** communicate to clients the importance of legal regulations in a clinical scenario	**4A.** demonstrate compliance with legal regulations and describe how to appropriately manage incidents of noncompliance<br><br>**4B.** communicate to clients the importance of legal regulations in a clinical setting

Examples of session-level/meeting-level/unit-level outcomes to use as a guide to fulfill **NGO 12.1**:

- explain the role of OSHA in regulating veterinary practices to ensure workplace safety (e.g., infectious, chemical and physical) for all employees,
- demonstrate compliance with OSHA regulations and recognize and describe how to appropriately manage incidents of noncompliance within a practice,
- recognize the need to obtain and maintain valid licensure for handling controlled substances,
- complete and submit health certificates and other regulatory forms according to the state and federal rules,
- identify the clinical signs, clinical course, transmission potential (including vectors), and pathogen associated with transboundary animal diseases,
- identify local and federal diseases of regulatory importance, including emerging and transboundary diseases, and report them to appropriate authorities (e.g., USDA, state animal health commission, and department of state health services),
- describe which drugs and uses of drugs are legal and illegal for use in Texas and the United States in common domestic species,1
- describe the sources that can be used to determine if a drug is legal for use in common domestic species at the state and federal levels,1
- read and interpret a drug label and apply the information to make a legal and clinically-appropriate drug decision,
- explain the process used to report adverse drug and vaccine reactions to the appropriate regulatory agency (e.g., EPA, USDA, FDA).

(cc) BY-NC This work is licensed under a Creative Commons Attribution-NonCommercial 4.0 International

NGO 13: Animal welfare

13.1 The graduate will apply the principles of animal welfare for the protection of animals through the prevention and relief of suffering and the humane and responsible management according to established codes of practice.

***** This rubric defines competencies for each NGO that students should complete in each year of the curriculum*****

Competency Rubric for each Year of the Curriculum	1VM	2VM	3VM	4VM
	Level of competence following completion of year 1.	*Level of competence following completion of year 2.*	*Level of competence following completion of year 3.*	*Expected level of competence at graduation.*
**13.1** Apply the principles of animal welfare for the protection of animals through the prevention and relief of suffering and the humane and responsible management according to established codes of practice.	**1A.** discuss the principals of animal welfare to the use of animals in teaching/research<br><br>**1B.** define principles of animal welfare	**2A.** recognize the signs of animal neglect and cruelty<br><br>**2B.** communicate principles of animal welfare to clients, healthcare team, and society in clinical scenarios<br><br>**2C.** discuss the principles of animal welfare to the use of animals in research	**3A.** explain the legal and ethical responsibilities of the veterinarian with regard to animal welfare in a clinical scenario, research setting, and society	**4A.** apply the principles of animal welfare for clinical patients and research animals<br><br>**4B.** explain the legal and ethical responsibilities of the veterinarian with regard to animal welfare<br><br>**4C.** communicate principles of animal welfare to clients, healthcare team, general public

Examples of session-level/meeting-level/unit-level outcomes to use as a guide to fulfill **NGO 13.1**:

- describe the role of the veterinarian in promoting the human-animal bond and communicate the importance of this relationship,
- describe common management systems for common domestic species and compare the advantages and disadvantages of each,
- communicate effectively to a client how to properly care for their animal and how to identify accurate sources of information,
- recognize the signs of animal neglect and cruelty and recognize the characteristics of animal hoarders and abusers and explain the legal and ethical responsibilities of the veterinarian in the identification, investigation, and prosecution of these cases,
- describe the Five Freedoms and their relevance to modern animal care,
- discuss the guiding principles for the ethical use of animals in research,
- describe the varying principles on animal rights,

cc BY-NC This work is licensed under a Creative Commons Attribution-NonCommercial 4.0 International

- describe environmental enrichment techniques for pets, livestock, laboratory animals, and zoo animals.

13.2 The graduate will apply animal restraint and handling techniques to safely and humanely work with patients and support clients and other members of the health care team.

***** This rubric defines competencies for each NGO that students should complete in each year of the curriculum*****

Competency Rubric for each Year of the Curriculum	1VM	2VM	3VM	4VM
	Level of competence following completion of year 1.	*Level of competence following completion of year 2.*	*Level of competence following completion of year 3.*	*Expected level of competence at graduation.*
**13.2** Apply animal restraint and handling techniques to safely and humanely work with patients and assist clients and other members of the health care team.	**1A.** describe methods of patient restraint for common domestic species<br><br>**1B.** perform restraint techniques safely and effectively	**2A.** perform restraint techniques safely and effectively	**3A.** select an appropriate restraint technique based upon the animal and situation<br><br>**3B.** perform animal restraint techniques safely and effectively in clinical scenarios	**4A.** apply animal restraint and handling techniques to safely and humanely work with patients and assist clients and other members of the health care team

Examples of session-level/meeting-level/unit-level outcomes to use as a guide to fulfill **NGO 13.2**:

- describe common methods of patient restraint for common domestic species,
- select an appropriate restraint technique based upon the animal and situation (e.g., disease state, clinical signs, animal compliance, environment, medical or surgical procedure, etc.),
- perform all widely accepted animal restraint techniques safely and effectively on all common domestic species,
- describe the precepts of animal welfare and explain how animal welfare relates to animal restraint,
- apply knowledge of animal behavior to ensure patient safety and the safety of the health care team during animal restraint.

BY-NC This work is licensed under a Creative Commons Attribution-NonCommercial 4.0 International

13.3 Determine when euthanasia is an appropriate option for a patient or population, effectively communicate the option with owners, and describe how to perform euthanasia safely, effectively and humanely

***** This rubric defines competencies for each NGO that students should complete in each year of the curriculum*****

Competency Rubric for each Year of the Curriculum	1VM	2VM	3VM	4VM
	Level of competence following completion of year 1.	*Level of competence following completion of year 2.*	*Level of competence following completion of year 3.*	*Expected level of competence at graduation.*
**13.3** Determine when euthanasia is an appropriate option for a patient, or population. Effectively communicate the option with owners, and describe how to perform euthanasia.		**2A.** describe ethical methods of euthanasia<br><br>**2B.** identify resources available for clients regarding euthanasia<br><br>**2C.** explain AVMA-approved methods of euthanasia	**3A.** perform euthanasia techniques for common domestic species in a clinical scenario<br><br>**3B.** create criteria to assist clients when electing euthanasia to alleviate suffering<br><br>**3C.** provide appropriate support to clients before, during, and after euthanasia of the animal in a clinical scenario	**4A.** select appropriate euthanasia technique for a clinical patient<br><br>**4B.** provide appropriate support to clients before, during, and after euthanasia of the animal

Examples of session-level/meeting-level/unit-level outcomes to use as a guide to fulfill **NGO 13.3**:

- create a set of criteria that can help a client decide when it is appropriate to euthanize an animal to alleviate its suffering,
- perform euthanasia on all common domestic species safely, effectively, and humanely,
- locate and utilize the AVMA Guidelines for Euthanasia,
- recognize the various expressions of human grief and be able to effectively mediate client reactions during euthanasia,
- provide appropriate support to clients before, during, and after euthanasia of the animal.

(cc) BY-NC This work is licensed under a Creative Commons Attribution-NonCommercial 4.0 International
