# Supplementary material for: Identifying information literacy skills and behaviors in the curricular competencies of health professions
Source: J Med Libr Assoc. 2020 Jul 1;108(3):463–79. doi: 10.5195/jmla.2020.833 (PMC7441914; doi:10.5195/jmla.2020.833)
Supplement: Supplementary file 4 — Appendix D: Texas A&M University Irma Lerma Rangel College of Pharmacy: Professional competencies [file jmla-108-3-463-s04.pdf]

JMLA Journal of the Medical Library Association

Supplemental content to
J Med Libr Assoc. Jul;108(3):dx.doi.org/10.5195/jmla.2020.833
www.jmla.mlanet.org
© Waltz, Moberly, Carrigan 2020

Identifying information literacy skills and behaviors in the curricular competencies of health professions

Micah J. Waltz; Heather K. Moberly, AHIP; Esther E. Carrigan, AHIP

APPENDIX D

Texas A&M University Irma Lerma Rangel College of Pharmacy: Professional competencies

TAMHSC DENTISTRY MEDICINE NURSING PUBLIC HEALTH Search

Irma Lerma Rangel PHARMACY TEXAS A&M UNIVERSITY

About COP Administration Academics Directory Research Giving Newsstand

Professional Competencies

Home » Current Students » Professional Competencies

Upon graduation, students will be able to:

- Provide comprehensive patient-centered care by designing, implementing, evaluating and continually refining pharmacy care plans based on best pharmacotherapy practices that incorporate health literacy, cultural competence, and psychosocial and socioeconomic factors to optimize patient outcomes.
- Provide evidence-based care to populations through disease management programs and protocols that are derived from analysis of epidemiologic and pharmacoeconomic data, medication use criteria, medication use review and risk reduction strategies.
- Promote public health through health literacy, health improvement, wellness and disease prevention strategies.
- Enhance the quality of population care by participating in the development of medication use and health promotion policies and by contributing to the accessibility and utilization of effective health care services.
- Communicate and collaborate effectively both verbally and in writing to engender a team approach to health care.
- Access, analyze and interpret relevant resources to provide comprehensive drug information to patients and health care providers on the safe and effective use of medications.
- Perform duties in accordance with legal, ethical and professional standards, and accept personal responsibility and accountability for one's actions.
- Maintain professional competence by identifying and evaluating emerging issues, trends, products and services that may impact patient and population outcomes, management of resources and medication use systems, disease prevention services and public health policy.
- Utilize and manage human, physical, medical, informational and technological resources to ensure accurate, safe, efficient and cost-effective drug distribution, medication use, and patient care.
- Engage with and manage medication use systems to assure patient safety by applying patient- and population-specific data, quality assurance strategies and research processes to optimize patient outcomes and minimize medication-related adverse events and errors.

Approved April 27, 2011

Howdy · Directory · Maps · Jobs · Email · IT

Current Students

- Contact information update
- Directory
- Criminal Background Check and Drug Screening
- Curriculum
- › *Professional Competencies*
- Portfolios
- Academic Calendar 2016-2017
- Doctorate of Pharmacy program
- Scholarships
- Dual Degree - Pharm.D./MBA
- Library and Learning Resources
- Financial Aid
- Registrar
- Applying to Residency Programs
- E*Value
- Problem Early Alert Network
- Project Requests
- Policies +
- Class of 2017 Graduation
- Academics
- Student Handbook +
- organizations +
- International Students
- File a complaint with ACPE

Quick Links

- Office of the Dean
- Academic Affairs
- Student Affairs
- Pharmaceutical Sciences
- Pharmacy Practice
- Experiential Education
- Finance & Administration
- College Events
- Employment Opportunities
- Room Reservations
- Strategic Plan
- Incident & Accident Reporting
- Human Resources

Information

- Contact Us
- FAQs
- Press & Media
- Contact Webmaster
- IT Help Desk
- HSC Alert

Affiliations

MEMBER OF THE TEXAS A&M UNIVERSITY SYSTEM

AACP

ACPE

Texas A&M Irma Lerma Rangel College of Pharmacy
1010 W. Avenue B
Kingsville, TX 78363
361-221-0604 | FAX 361-221-0790

State of Texas · Texas Homeland Security · Public Information Act · Risk, Fraud & Misconduct Hotline · Statewide Search · State Link Policy
Security · Equal Opportunity/Nondiscrimination · Employment Opportunities · Texas Veterans Portal · Site Policies · Web Accessibility
