# Supplementary material for: Identifying information literacy skills and behaviors in the curricular competencies of health professions
Source: J Med Libr Assoc. 2020 Jul 1;108(3):463–79. doi: 10.5195/jmla.2020.833 (PMC7441914; doi:10.5195/jmla.2020.833)
Supplement: Supplementary file 5 — Appendix E: Texas A&M University School of Public Health: Competencies [file jmla-108-3-463-s05.pdf]

JMLA Journal of the Medical Library Association

Supplemental content to
J Med Libr Assoc. Jul;108(3):dx.doi.org/10.5195/jmla.2020.833
www.jmla.mlanet.org
© Waltz, Moberly, Carrigan 2020

Identifying information literacy skills and behaviors in the curricular competencies of health professions

Micah J. Waltz; Heather K. Moberly, AHIP; Esther E. Carrigan, AHIP

APPENDIX E

Texas A&M University School of Public Health: Competencies

TAMHSC DENTISTRY MEDICINE NURSING PHARMACY PUBLIC HEALTH Search

PUBLIC HEALTH
TEXAS A&M UNIVERSITY

About SPH | Degrees | Departments | Campuses | Research | Administration | Climate & Diversity | Contact Us | Giving | Newsstand

Competencies

All competencies should be considered updated for 2018-2019. Please access them here: http://public-health.tamu.edu

Home » Students » Student Handbook » Competencies

SPH's core curriculum, outlined across six core courses, is based on public health competencies which are in keeping with the Association of Schools of Public Health (ASPH) and the Council on Education for Public Health (CEPH).

Core Competencies

[Updated in Summer 2013]

Biostatistics Competencies

- Describe the roles biostatistics serves in the discipline of public health.
- Describe basic concept of probability, random variation and commonly used statistical probability distributions.
- Describe preferred methodological alternatives to commonly used statistical methods when assumptions are not met.
- Distinguish among the different measurement scales and the implications for selection of statistical methods to be used based on these distinctions.
- Apply descriptive techniques commonly used to summarize public health data.
- Apply common statistical methods for inference.
- Apply descriptive and inferential methodologies according to the type of study design for answering a particular research question.
- Apply basic informatics techniques with vital statistics and public health records in the description of public health characteristics and in public health research and evaluation.
- Interpret results of statistical analyzes found in public health studies.
- Develop written and oral presentations based on statistical analyses.
- Define "rural" status.
- Use appropriate statistical analysis to compare and contrast sociodemographic characteristics of rural, suburban, and urban populations.
- Use appropriate statistical analysis to describe unique risk factors related to disease in rural area.
- Identify challenges inherent in conducting research on rural populations.

Howdy · Directory · Maps · Jobs · Email · IT

Student Handbook
- Introduction and Disclaimer
- Additional Services
- Attendance & Holidays
- Awards & Honors
- Change in Program Status
- › *Competencies*
- Course Related Processes
- Culminating Experiences
- Grades & Academic Standing
- Professionalism
- Program Overview
- Student Life Special Items
- Student Organizations
- Appendix I - Contact Information
- Student Complaints
- Student Grievances and Appeals

Environmental Health Competencies

- Describe the direct and indirect human, ecological and safety effects of major environmental and occupational agents.
- Describe genetic, physiologic and psychosocial factors that affect susceptibility to adverse health outcomes following exposure to environmental hazards.
- Describe federal and state regulatory programs, guidelines and authorities that control environmental health issues.
- Specify current environmental risk assessment methods.
- Specify approaches for assessing, preventing and controlling environmental hazards that pose risks to human health and safety in both urban and rural settings.
- Explain the general mechanisms of toxicity in eliciting a toxic response to various environmental exposures.

- Discuss various risk management and risk communication approaches in relation to issues of environmental justice and equity.

Epidemiology Competencies

- Identify key sources of data for epidemiologic purposes.
- Identify the principles and limitations of public health screening programs.
- Describe a public health problem in terms of magnitude, person, time and place.
- Explain the importance of epidemiology for informing scientific, ethical, economic and political discussion of health issues.
- Comprehend basic ethical and legal principles pertaining to the collection, maintenance, use and dissemination of epidemiologic data.
- Apply the basic terminology and definitions of epidemiology.
- Calculate basic epidemiology measures.
- Communicate epidemiologic information to lay and professional audiences.
- Draw appropriate inferences from epidemiologic data.
- Evaluate the strengths and limitations of epidemiologic reports.
- Define “rural” status.
- Compare and contrast sociodemographic characteristics of rural, suburban, and urban populations.
- Describe unique risk factors related to disease in rural areas.
- Discuss accessibility to medical care in rural areas and the impact this has on rates of disease.
- List the five leading adverse health outcomes in rural, suburban, and urban areas.
- Identify challenges inherent in conducting research on rural populations.
- Identify data sources that are especially useful in describing the health status of rural populations.

Health Promotion and Community Health Sciences

- Identify basic theories, concepts and models from a range of social and behavioral disciplines that are used in public health research and practice.
- Identify the causes of social and behavioral factors that affect health of individuals and populations.
- Identify individual, organizational and community concerns, assets, resources and deficits for social and behavioral science interventions.
- Identify critical stakeholders for the planning, implementation and evaluation of public health programs, policies and interventions.
- Describe steps and procedures for the planning, implementation and evaluation of public health programs, policies and interventions.
- Describe the role of social and community factors in both the onset and solution of public health problems.
- Describe the merits of social and behavioral science interventions and policies.
- Apply evidence-based approaches in the development and evaluation of social and behavioral science interventions.
- Apply ethical principles to public health program planning, implementation and evaluation.
- Specify multiple targets and levels of intervention for social and behavioral science programs and/or policies.
- Define concept of rurality.
- Explain unique circumstances related to public health in rural areas.
- Explain how public health service delivery in rural areas differs from that in urban and suburban areas.

Health Policy and Management

- Articulate a definition of public health that captures the unique characteristics of the field (e.g., population-focused, community-oriented, prevention-motivated and rooted in social justice) and how these contribute to professional practice.
- Identify the main components and issues of the organization, financing and delivery of health services and public health systems in the U.S.
- Describe the legal basis for public health and health services.
- Describe the attributes of ethical leadership in public health and demonstrate leadership skills for building partnerships.
- Communicate public health policy and management issues using appropriate channels and technologies and effective written and oral skills.
- Describe the principles of program planning, development, management and evaluation in public health initiatives.
- Apply an understanding of feedback loops to public health dynamics.
- Apply the core functions of assessment, policy development, and assurance in the analysis of public health problems and their solutions.

Concentration Competencies

Biostatistics Concentration Competencies (MPH)

- Translate a study's scientific questions or aims into testable statistical hypotheses and propose appropriate statistical methods to test those hypotheses.
- Perform sample size and power calculations for standard study designs to ensure that a study is sufficiently powered for achieving scientific aims or testing a specific research hypothesis.
- Describe the strengths and weaknesses of commonly used study designs and the data collection methods that go with these designs.
- Perform analyses of stated hypotheses using a variety of analytical tools including analysis of variance, multiple regression, nonparametric statistics, logistic regression, and methods for analyzing failure time data, and correlated data.
- Communicate commonly used statistical ideas and methods to collaborators in non-technical terms, both orally and in writing.

Biostatistics Concentration Competencies (MSPH)

- Use a range of tools in analytical problem solving, decision making, and evaluation in public health issues.
- Demonstrate an understanding of statistical theory and its application.
- Apply biostatistical methods to public health data.
- Apply biostatistical methods in collaboration with other health professionals.
- Define "rural" status.
- Use appropriate statistical analysis to compare and contrast sociodemographic characteristics of rural, suburban and urban populations.
- Use appropriate statistical analysis to describe unique risk factors related to disease in rural area.
- Identify challenges inherent in conducting research on rural populations.

Environmental Health Concentration Competencies (MPH)

- Develop and implement strategies for mitigating environmental health hazards.
- Describe the synergistic and multiplicative factors that influence solutions for environmental health hazards.
- Describe the process in which policies are developed and implemented to handle and reduce environmental health risks and hazards.
- Apply the current approaches for risk assessment and risk communication.
-
- Environmental Health Concentration Competencies (MSPH)
- Acquire knowledge of the context of environmental health.
- Apply theoretical and conceptual models and skills relevant to the discipline.
- Describe the strengths and weaknesses of study designs to appropriately address specific research questions in the field.
- Collect primary data or assemble and manage existing data from public and private source.
- Explain procedures that ensure the reproducibility of the science, the responsible use of resources, the ethical treatment of research subjects.
- Demonstrate proficiency in the appropriate application of analytical techniques to evaluate research questions.
- Effectively communicate the process and findings of research.

Occupational Health Concentration Competencies (MSPH)

- Acquire knowledge of the context of occupational health.
- Apply theoretical and conceptual models and skills relevant to the discipline.

- Describe the strengths and weaknesses of study designs to appropriately address specific research questions in the field.
- Collect primary data or assemble and manage existing data from public and private sources.
- Explain the procedures that ensure the reproducibility of the science, the responsible use of resource, the ethical treatment of research subjects.
- Demonstrate proficiency in the appropriate application of analytical techniques to evaluate research questions.
- Effectively communicate the process and findings of research.

Epidemiology Concentration Competencies (MPH)

- Understand and recognize how epidemiologic methods are tailored to address the unique challenges presented by epidemiologic specialty/content areas (e.g., infectious disease, chronic disease, social, occupational, environmental, spatial, disaster, and injury).
- Describe the extent to which common epidemiologic study designs and biostatistical methods can or cannot provide evidence for causal relationships.
- Apply descriptive and analytical data analysis methods and data management techniques to epidemiologic data.
- Prepare abstracts, presentations, and/or reports in the format used for scientific or public meetings, peer-reviewed journals, or public health agencies.

Epidemiology Competencies (MSPH)

- Define, assess and understand the health status of populations, determinants of health and illness, and factors contributing to health promotion and decease prevention.
- Apply data collection and maintenance processes and computer systems storage/retrieval strategies.
- Discuss the role of epidemiology in developing, implementing and evaluating public health policy.
- Identify the vectors and characteristics of the most important common infectious diseases and predictors of the most common chronic disease.
- Define “rural” status.
- Compare and contrast sociodemographic characteristics of rural, suburban, and urban populations.
- Describe unique risk factors related to disease in rural areas.
- Discuss accessibility to medical care in rural areas and the impact this has on rates of disease.
- List the five leading adverse health outcomes in rural, suburban, and urban areas.
- Identify challenges inherent in conducting research on rural populations.
- Identify data sources that are especially useful in describing the health status of rural populations.

Health Promotion and Community Health Sciences concentration competencies (MPH)

- Describe the steps that are necessary in planning applied research as it relates to specific public health issues.
- Select and explain a research design that is appropriate for examining a particular public health research question at multiple levels.
- Integrate multiple methodologies by demonstrating the use of “methodological triangulation” (use of multiple methods) and a systems approach to study a single public health problem or program.
- Conduct ethnographic research.
- Design and administer a variety of surveys.
- Develop research proposals using a variety of research methods.
- Compare and contrast varying approaches to defining community and community assessment.
- Identify community assets and resources.
- Identify primary and secondary data sources useful in community assessment.
- Use community health status information to establish priorities.
- Plan, conduct, and report on a community health status assessment.
- Identify community characteristics and related data from a community assessment that would influence how health improvement interventions should be planned for that community.

- Generate a well-written program plan that identifies and describes the epidemiology of a health problem, identifies and documents the causes of the problem, incorporates a social-ecological analysis of approaches to solve the problem, explains how social and behavioral concepts and theories can be applied to solve the problem, incorporates an acceptable and defined logical structure, demonstrates cultural appropriateness, and analyzes ethical concerns in planning and implementation.
- Design and defend a budget for a public health intervention.
- Demonstrates the ability to be a productive, contributing and effective member of a planning group.
- Demonstrate the ability to collect and use appropriate information for program planning, implementation and both process and outcome evaluation activities.
- Recognize personal, professional and social value and how they interact in the planning, implementation and evaluation of public health programs.
- Design and implement individual, group, organizational and community assessments to identify public health related assets, resources, needs, and concerns.
- Specify multiple targets and levels of intervention for social and behavioral science programs and/or policies.
- Demonstrate the capability to develop a theory-based and practical intervention plan for addressing individual, group, organization or community public health needs.
- Employ effective communication strategies and skill.
- Demonstrate the ability to work effectively with diverse populations.
- Describe major philosophical orientations and types of evaluation.
- Draft a budget for a public health program.
- Describe the evaluation process recommended by the CDC and the Kellogg Foundation.
- Write, analyze, and evaluate evaluation questions; that is, analyze evaluation issues and write an evaluation.
- Critically evaluate study design and statistics employed.
- Describe the meaning and measurement of reliability and validity.
- Analyze effects in process and impact evaluations.
- Describe how to increase the usability of an evaluation.

Health Promotion and Community Health Sciences (MPH in Border Health)

- Identify basic theories, concepts and models from a range of social and behavioral disciplines that are used in public health research and practice.
- Identify the causes of social and behavioral factors that affect health with particular emphasis in border populations.
- Identify individual, organizational and community concerns, assets, resources and deficits for social and behavioral science interventions.
- Identify critical stakeholders for the planning, implementation and evaluation of public health programs, policies and interventions in border health settings.
- Apply systems thinking as an important analysis perspective in the context of border public health.
- Describe comparatively health care and public health system internationally.
- Identify justice and equity issues in border health.
- Describe steps and procedures for the planning, implementation and evaluation of public health programs, policies and interventions.
- Describe the role of social and community factors in both the onset and solution of public health problems.
- Identify social and behavioral issues relevant to public health in the United States and the U.S. Mexico border region.
- Describe the merits of social and behavioral science interventions and policies.
- Apply evidence-based approaches in the development and evaluation of social and behavioral science interventions.
- Apply ethical principles to public health program planning, implementation and evaluation.
- Specify multiple targets and levels of intervention for social and behavioral science programs and policies in border settings.
- Define concept of rurality.
- Explain unique circumstances related to public health in rural areas.
- Explain how public health service delivery in rural areas differs from that in urban and suburban

Health Promotion and Community Health Sciences concentration competencies (MSPH)

- Recognize personal, professional and social values and how they interact in the planning, implementation and evaluation of public health programs in rural and urban areas; and demonstrate professionally ethical practices.
- Employ effective verbal and written communication strategies and skills.
- Demonstrate the ability to work effectively with diverse and/or rural populations.
- Design and implement individual, group, organizational, and community assessments to identify public health related assets, resources, needs and concerns.
- Demonstrate the capability to develop a theory-based and practical intervention plan for addressing individual, group, organization or community public health needs.
- Demonstrate the capacity to implement a program plan for addressing public health issues in rural and underserved areas.
- Demonstrate the ability to collect and use appropriate information for program planning, implementation and both process and outcome evaluation activities.
- Design, execute, report, and defend a publishable thesis research project.
- Define concept of rurality.
- Explain unique circumstances related to public health in rural areas.
- Explain how public health service delivery in rural areas differs from that in urban and suburban areas.

Health Policy and Management Concentration Competencies (MPH)

- Articulate a set of core values and an achievable mission and vision.
- Apply principles of strategic planning and marketing to public health.
- Describe the legal basis for public health and health services.
- Understand the policy processes in the United States, and apply this knowledge to the formation public health policy.
- Write clear and concise public health policy recommendations drawing effectively on peer-reviewed literature.
- Understand how state and federal laws affect the operation of health plans and health facilities.
- Understand the basic public health finance and accounting principles and their application.
- Apply basic economic principles to predict supply and demand of public health services.

Health Policy and Management Concentration Competencies (MSPH)

- Develop and apply public health principles including statistical reasoning and methods in addressing, analyzing and solving problems in public health; health care; and biomedical, clinical and population-based research.
- Define, explain and apply the key concepts in their field of specialization.
- Formulate a research hypothesis, and create the study aims necessary to address the hypothesis; determine the appropriate study design to address the hypothesis; have sufficient understanding of the public health outcomes to measure them in a population setting; review the existing research literature; write a research protocol that addresses how to approach the problem; use existing data sets including vital statistics records, disease registries, and clinic and hospital records; use data management and statistical software to analyze the basic relationships between risk factors and outcome data; interpret and present the results; and write the results in for publication in a report or professional journal.
- Collect, manage and organize data to produce information that is disseminated; to gather, process, and present information to different audiences in-person, through information technologies, or through media channels; and to strategically design the information and knowledge exchange process to achieve specific objectives.
- Recognize ethical issues and behave in accordance with ethical standards of conduct in the design, implementation, analysis, and dissemination of scientific research.

Health Policy and Management Concentration Competencies (MHA)

- Accountability-Communicates requirements and expectations, sets limits, demands high performance, confronts performance problems, creates culture of accountability.
- Analytical Thinking-Breaks down problems, identifies basic relationships, develops complex plans or analyses.
- Community Orientation-responds appropriately to community needs, maintains clear communication, takes personal responsibility for initiating collaborative planning, participates with and understands the community, provides services to the community, advocates for the broader health environment.
- Financial Skills-Explains the organization's financial metrics and reports, manages budgets and assets, understands impact of reimbursement models, evaluates financial analyses and investments, develops long-term financial plans.
- Information Seeking-consults available resources, investigates beyond routine questions, delves deeper, conducts research to

maintain knowledge, is recognized as a user of best practices.

- Organizational Awareness-Uses formal structure, applies understanding of informal structure, adapts actions to climate and culture, considers priorities and values of multiple constituencies, uses insights of stakeholders underlying actions and issues.
- Performance Measurement-monitors indicators of performance, monitors a scorecard of quantitative and qualitative measures, uses evidence based approaches to support community awareness.
- Team Leadership-manages team meetings well, keeps people informed, promotes team effectiveness, obtains resources/takes care of the team, demonstrates leadership, is a role model for leadership.

Dr.PH in Epidemiology and Environmental Health Competencies

- Evaluate epidemiologic and environmental health evidence pertaining to the scope and magnitude of environmental threats to public health.
- Plan, implement and evaluate interventions designed to respond to environmental hazards that threaten individual, community and population health.
- Articulate conceptual basis for understanding the context and key factors associated with health and public health problems.
- Explain the theoretical foundations and change strategies for addressing critical public health issues.
- Relate epidemiology and environmental health issues to at least one other public health discipline.
- Prepare reports and scholarly presentations and participate in conference presentations in order to appropriately influence relevant state-of-the-art practice.
- Work in interdisciplinary teams and develop campus-community partnerships.
- Submit grant applications to support proposed research and practice activities.

Dr.PH. in Health Promotion and Community Health Sciences

- Plan, implement and evaluate interventions designed to improve individual, community and population health status.
- Clearly, articulate conceptual basis for understanding the context and key factors associated with health and public health problems.
- Identify breadth social and behavioral determinants of health and public health problems.
- Understand the theoretical foundations and change strategies for addressing critical public health issues.
- Relate social and behavioral health issues to at least one other public health discipline.
- Demonstrate research skills in identifying problem, delineating theory of the problem, selecting appropriate design and analytical approaches, analyzing data, and interpreting results.
- Prepare reports and scholarly presentations and participate in conference presentations in order to appropriately influence relevant state-of-the-art practice.
- Work in interdisciplinary teams and develop campus-community partnerships.
- Assume professional research and practice roles in health promotion and disease prevention, community development, program planning and evaluation.
- Submit grant applications to support proposed research and practice activities
- Define concept of rurality.
- Explain unique circumstances related to public health in rural areas.
- Explain how public health service delivery in rural areas differs from that in urban and suburban areas.

Ph.D. in Health Services Research

- Identify and assemble a large body of existent research addressing a specific research agenda.
- Determine the quality of the work represented by that body of published research.
- Articulate problem specification and theoretical paradigms appropriate to a specific research agenda.
- Articulate subsequent researchable hypotheses that might further our knowledge base regarding the specific topic.
- Conceive of a research design that would allow these hypotheses to be tested in a manner that would stand up to peer review.
- Integrate interdisciplinary and multidisciplinary contributions to a specific research agenda.
- Develop measurement tools necessary to implement the selected project design.
- Design the analytical steps necessary to maximize the applicability of the data available to the research questions identified.
- Organize a team of colleagues necessary to undertake the data gathering and analytical tasks.

- Achieve mastery of basic statistical and epidemiological computation techniques.
- Execute quantitative and qualitative analytical techniques appropriate to the research design and data studied.
- Draw appropriate conclusions about the research undertaken.
- Prepare appropriate reports and scholarly publications and participate in conference presentations in order to appropriately influence relevant state-of-the-art practice.

Texas A&M School of Public Health
212 Adriance Lab Road
College Station, TX 77843-8371
979-436-9443 | Fax 979-436-9599
