# Supplementary material for: Identifying information literacy skills and behaviors in the curricular competencies of health professions
Source: J Med Libr Assoc. 2020 Jul 1;108(3):463–79. doi: 10.5195/jmla.2020.833 (PMC7441914; doi:10.5195/jmla.2020.833)
Supplement: Supplementary file 6 — Appendix F: Context of project and coding rubric [file jmla-108-3-463-s06.pdf]

JMLA Journal of the Medical Library Association

SC
Supplemental content to
J Med Libr Assoc. Jul;108(3):dx.doi.org/10.5195/jmla.2020.833
www.jmla.mlanet.org
© Waltz, Moberly, Carrigan 2020

Identifying information literacy skills and behaviors in the curricular competencies of health professions

Micah J. Waltz; Heather K. Moberly, AHIP; Esther E. Carrigan, AHIP

APPENDIX F
Context of project and coding rubric

We are evaluating how the Texas A&M University Medical Sciences Library (MSL) supports the development of information-related competencies from the five colleges with professional programs (College of Medicine, College of Nursing, College of Veterinary Medicine, Irma Lerma Rangel College of Pharmacy, and School of Public Health).

This evaluation begins with a multiyear multiphase data generation process:

- The first phase consists of initial coding of all five schools' competencies into a short list of pre-populated categories.
- The second phase is an additional round of categorization where the competencies are broken into subcategories generated from the competencies.
- The third phase is a multitiered approach where the coded competencies are:
    1. Compared to published national and international standards for each professional program.
    2. Evaluated with the curriculum committees of the respective programs to capture the intention for each information-related competencies.
    3. Aligned across the five curricula using the generated data from the first two phases to identify inter-curricular core units of instruction.

Interact with the faculty in each curriculum who teach classes with designated competencies to learn how they interpret and teach their information-related competencies.

After these data have been generated, an evaluation of the current MSL educational practices supporting the five professional programs will occur. Discrepancies between the interpretation of the information-related competencies in the classroom AND how MSL is supporting the development of these information-related competencies will be identified. Once the competencies have been identified, instruction supporting the development of the information-related competencies for each college will be either be redesigned or created. This instruction will be developed considering the aligned inter-curricula information-related competencies across the colleges' curricula to promote sustainable educational support from MSL faculty.

Purpose of coding

This round of coding is the first phase, which consists of the initial coding of the competencies from the five colleges with professional programs (Medicine, Nursing, Veterinary Medicine, Pharmacy, and Public Health) that MSL supports. This should be a "quick" first round of coding to categorize the competencies into predetermined core areas. This first round of coding is NOT based on individual aspects of competencies that are written with multiple parts such as pharmacy and veterinary medicine competencies may be (Example 1).

JMLA Journal of the Medical Library Association

SC
Supplemental content to
J Med Libr Assoc. Jul;108(3):dx.doi.org/10.5195/jmla.2020.833
www.jmla.mlanet.org
© Waltz, Moberly, Carrigan 2020

Coding instructions

To code a competency, identify the action verb of the competency—this may be tricky because some competencies have multiple clauses—and use the direct object of the verb as context for identifying the core area of each competency. For the more complicated competencies that include multiple elements, focus on the overall theme of the competency for this round of categorizing them (Example 1).

Example 1

From the TAMU Pharmacy competencies

1. Enhance the quality of population care by participating in the development of medication use and health promotion policies and by contributing to the accessibility and utilization of effective health care services.

All competencies must be coded. If there is a competency that does not appear to fit in the predetermined categories, code it as "Other" (Example 2).

Example 2

From the TAMU Medicine competencies

2. Demonstrate an awareness of leadership roles in medicine and society

Disagreements about coding

We will meet and discuss any discrepancies in our coding. If we mediate on how any competency should be coded, we will vote with a simple two-thirds majority ruling how that competency should be coded. If we find a situation where voting will not solve how to identify the most appropriate categorization (we all have a different code that we believe is most valid), the final decision about categorization will be done by someone who is not involved in the coding process (Carrigan).

Notes

With the Veterinary Medicine competencies, only code the performance indicators—do not code the 1VM, 2VM, etc. (specific year) competencies.

JMLA Journal of the Medical Library Association

SC
Supplemental content to
J Med Libr Assoc. Jul;108(3):dx.doi.org/10.5195/jmla.2020.833
www.jmla.mlanet.org
© Waltz, Moberly, Carrigan 2020

Coding rubric

Preliminary code categories	Coding question	Inclusion criteria	Exclusion criteria	Exclusion criteria example
Clinical Skills	Is *treatment* OR *diagnosis* OR *providing care* the overarching theme of the competency?	[action verb]+description about or use of medical tests/procedures, development of patient plans, physical examinations/palpations, use of collected information/data to inform diagnosis, and phrases such as "provide health OR palliative care."	If the medical tests or procedures are NOT the focus and are being used FOR an application that would be categorized in a different core area	Demonstrate understanding of epidemiology of common diseases within a population and the approaches which are useful in reducing their incidence and prevalence<br><br>**TAMU Medical School Competencies*
Communication Skills/Human Interaction	Is *communication* the overarching theme of the competency?	[action verb]+description about communication using any form of media including charting, collecting information from a population, or considering/accounting for/communicating with various populations (ethnic, cultural, religious, education, etc.).	NA	NA
Didactic Knowledge and Understanding	Is *gaining* or *demonstrating* ***knowledge*** the overarching theme of the competency?	[action verb about understanding/knowing/comprehending/integrating knowledge]+description about the concept—or group of concepts—that are expected to be understood	If the knowledge is being used to:<br>1. communicate a diagnosis<br>2. inform communication<br>3. diagnose,<br>4. inform policy/legalities	Demonstrate knowledge of common societal problems such as domestic societal problems such as domestic violence and substance abuse, including diagnosis, preventing, reporting, and treatment<br><br>**TAMU Medical School Competencies*

JMLA Journal of the Medical Library Association

SC
Supplemental content to
J Med Libr Assoc. Jul;108(3):dx.doi.org/10.5195/jmla.2020.833
www.jmla.mlanet.org
© Waltz, Moberly, Carrigan 2020

Preliminary code categories	Coding question	Inclusion criteria	Exclusion criteria	Exclusion criteria example
Information Seeking Behavior and Skills	Is *obtaining* or *evaluating* ***information*** the overarching theme of the competency?	[action verb about evaluating/analyzing/appraising/ critiquing/searching]+description about information appraised, evaluated, or sought out.	If the evaluation/ appraisal/critique is specific to statistics or study design.	Utilize information technology in providing medical care for individuals<br><br>**TAMU Medical School Competencies*
Legal Awareness, Application, organizational awareness and Advocacy/ Ethics	Is *awareness* of, *use* of, or *development* of **laws**, **policies**, or **organizational structure** the overarching theme of the competency?	[action verb about knowledge of, awareness of, identification of, or application of]+description of laws, policies, licensing boards, organizational structures, or development of policies/laws.	Use of didactic knowledge to explain the importance of policy.	Apply the principles of animal welfare for the protection of animals through the prevention and relief of suffering and the human and responsible management according to the established codes of practice<br><br>**TAMU Vet Med School Competencies*
Other	Is the overarching theme one that does not fall into one of the six pre-selected categories, for example *leadership*.	Any competency that does fall within the bounds of the described categories.	Any competency that can be included into one of the provided categories.	

JMLA Journal of the Medical Library Association

Supplemental content to
J Med Libr Assoc. Jul;108(3):dx.doi.org/10.5195/jmla.2020.833
www.jmla.mlanet.org
© Waltz, Moberly, Carrigan 2020

Preliminary code categories	Coding question	Inclusion criteria	Exclusion criteria	Exclusion criteria example
Statistics/ Experimental Design understanding and application	Is the *application, evaluation,* or *understanding* of ***statistics*** or ***experimental design*** the overarching theme of the competency?	[action verb about the description of, application of, understanding of, interpretation of OR analysis of/interpretation of]+statistical analyses, inferences, statistical techniques, basic statistical concepts such as probability, description of data, or experimental design	Communication about<br>1. statistics or experimental design<br>2. how statistics are used in a didactic discipline that is not a variant form of statistics, for example biostatistics	Describe the role biostatistics serves in the discipline of public health<br>**TAMU Public Health Competencies*
